# Bioadhesive Hydrogels for Tissue Repair: Design Strategies, Adhesion Mechanisms, and Emerging Applications

**DOI:** 10.3390/molecules31173085

**Published:** 2026-09-02

**Authors:** Seoha Kim, Hyejin Jo, Seunghun S. Lee

**Affiliations:** Department of Biomedical Engineering, Dongguk University, Seoul 04620, Republic of Korea; 2023111715@dgu.ac.kr (S.K.); annajo@dgu.ac.kr (H.J.)

**Keywords:** bioadhesive hydrogel, tissue adhesive, wound healing, tissue engineering, wet adhesion

## Abstract

Bioadhesive hydrogels combine tissue-adhesive properties with therapeutic multifunctionality, offering promising solutions for regenerative medicine. This comprehensive review examines the design strategies, fundamental adhesion mechanisms, and clinical applications of these biomaterials. We systematically discuss four primary adhesion mechanisms: physical interactions, chemical adhesion, topological mechanical interlocking, and bioinspired adhesion. Key design parameters, including wet-environment adhesion strength, self-healing capability, injectability, and controlled biodegradability, are analyzed and benchmarked against commercial products. Major material platforms, encompassing catechol-based systems, chitosan derivatives, gelatin/GelMA variants, Polyethylene glycol (PEG)-based adhesives, and multi-network hybrid systems, are evaluated for their adhesive performance and functional integration. Tissue-specific applications spanning wound healing, bone/cartilage repair, soft tissue sealing, vascular repair, and neural regeneration are critically assessed, emphasizing in vivo outcomes and clinical translation barriers. Finally, we discuss emerging frontiers, including artificial intelligence-guided material design, on-demand detachable adhesives, and regulatory pathways. Synthesizing over 140 peer-reviewed references from the past two decades, this review provides a systematic roadmap from fundamental adhesion science toward the clinical implementation of next-generation bioadhesive hydrogels.

## 1. Introduction

Tissue damage and surgical wounds represent a major clinical burden affecting millions of patients annually worldwide. The global tissue adhesive market was worth about USD 2.3 billion in 2023. It is projected to exceed USD 4.1 billion by 2030, which reflects rising demand for advanced wound closure technologies. Sutures and staples remain the standard closure methods, but they have clear drawbacks. They require invasive placements and carry risks of infection and scarring. They are also poorly suited to irregular defects and to delicate tissues such as vocal folds, neural tissue, and vascular structures. Although researchers have developed synthetic surgical adhesives, including cyanoacrylate-based glues and fibrin sealants, these products suffer from significant limitations. Cyanoacrylates generate toxic byproducts (formaldehyde upon degradation), exhibit brittle failure in dynamic environments, and provide inadequate wet adhesion. Fibrin glues are derived from blood plasma. They require complex preparation, have a limited shelf-life and carry a theoretical risk of pathogen transmission. Their adhesion strength is also modest, at about 10 kPa under physiological conditions [1,2]. Commercial tissue sealants such as CoSeal and DuraSeal offer improved biocompatibility but remain relatively rigid, limit tissue conformability, and cannot simultaneously provide adhesion with other therapeutic functions [3].

Hydrogels, cross-linked polymer networks with high water content (typically 70–99%), have emerged as a superior platform for addressing these clinical limitations. Hydrogels differ from rigid surgical adhesives in three ways. They conform to irregular tissue surfaces. Their elastic moduli of 0.1–1000 kPa approach those of native tissue. Their high water content mimics the extracellular matrix, which gives them excellent biocompatibility [4,5]. The seminal work by Li et al. demonstrated that hydrogels can achieve robust wet adhesion exceeding 100 kPa through carefully designed chemistry, fundamentally challenging the notion that adhesion in aqueous environments is inherently weak [6]. Subsequent innovations by Yuk and colleagues introduced the concept of dry double-sided tape hydrogels [7]. wherein water-repelling interfacial layers and absorptive bulk hydrogels that synergistically create strong, reversible adhesion, a principle inspired by everyday adhesive tapes but applied to biological tissues. More recently, Deng et al. demonstrated electrical bioadhesive interfaces achieving both mechanical adhesion and electrical signal transmission, opening possibilities for bioelectronic tissue integration [8].

The transformative advantage of hydrogel-based bioadhesives lies in their multifunctionality. Passive mechanical adhesives only hold tissue together. Bioadhesive hydrogels can be engineered to do far more. They can deliver therapeutic agents, promote tissue healing, respond to physiological stimuli, conduct electrical signals, prevent infection, and adapt to dynamic tissue motion [9,10]. This multifunctionality positions bioadhesive hydrogels as integrated therapeutic platforms rather than simple tissue-bonding agents. The clinical significance is underscored by the FDA/EMA approval of 28 injectable hydrogel-based products as of 2024, with over 400 active clinical trials involving hydrogel materials [11].

Scope and literature coverage. The literature examined here was identified through [Web of Science, Scopus and PubMed] using combinations of the terms “bioadhesive hydrogel”, “tissue adhesive”, “wet adhesion”, “catechol”, “self-healing hydrogel” and “tissue sealant”. The search window was [January 2005 to March 2025]. Priority was given to work published from 2019 onward, which accounts for approximately [70%] of the studies cited. Earlier papers were retained only where they established a mechanism that remains foundational, such as the first synthetic DOPA-functionalized polymers. We included peer-reviewed primary studies that reported at least one quantitative adhesion metric—lap shear strength, burst pressure or interfacial toughness—together with clinical reports on approved products. Conference abstracts, preprints and studies without quantitative adhesion data were excluded. The review is organized comparatively rather than as a catalogue: each platform is assessed against the same four criteria (wet adhesion strength, self-healing recovery, degradation control and evidence of in vivo durability), and we state explicitly where the available evidence is too heterogeneous to support a direct comparison.

This review comprehensively examines the state-of-the-art in bioadhesive hydrogel design, synthesizing insights from materials science, biochemistry, biomedical engineering, and clinical medicine. We structure the analysis around four dimensions. The first is the biochemical and physical mechanisms enabling tissue adhesion. The second is critical design parameters that balance adhesion strength, biocompatibility, and function. The third is the major material platforms and how they compare in terms of advantages. The fourth is tissue-specific applications with emphasis on translation barriers and regulatory pathways. Particular attention is given to developments from 2020 to 2025, reflecting the explosive growth of this field. Key references establishing foundational concepts include the pioneering work of Li et al. demonstrating tough adhesives [6], Yuk et al. introducing dry double-sided tape mechanics [7], Blacklow et al. describing mechanically active adhesive dressings [12], and recent data-driven hydrogel design approaches published in Nature [13]. Additionally, our laboratory has contributed to this field through development of self-healing bioadhesive tissue implants for vocal fold repair and augmentation [14], demonstrating clinical translation potential for soft tissue applications. This review encompasses over 140 peer-reviewed references, providing a systematic roadmap from fundamental adhesion science toward clinical implementation. We do not attempt to list every reported formulation. Instead we compare platforms against common criteria and identify where the evidence is weak, inconsistent or absent.

## 2. Adhesion Mechanisms

**Figure 1:**
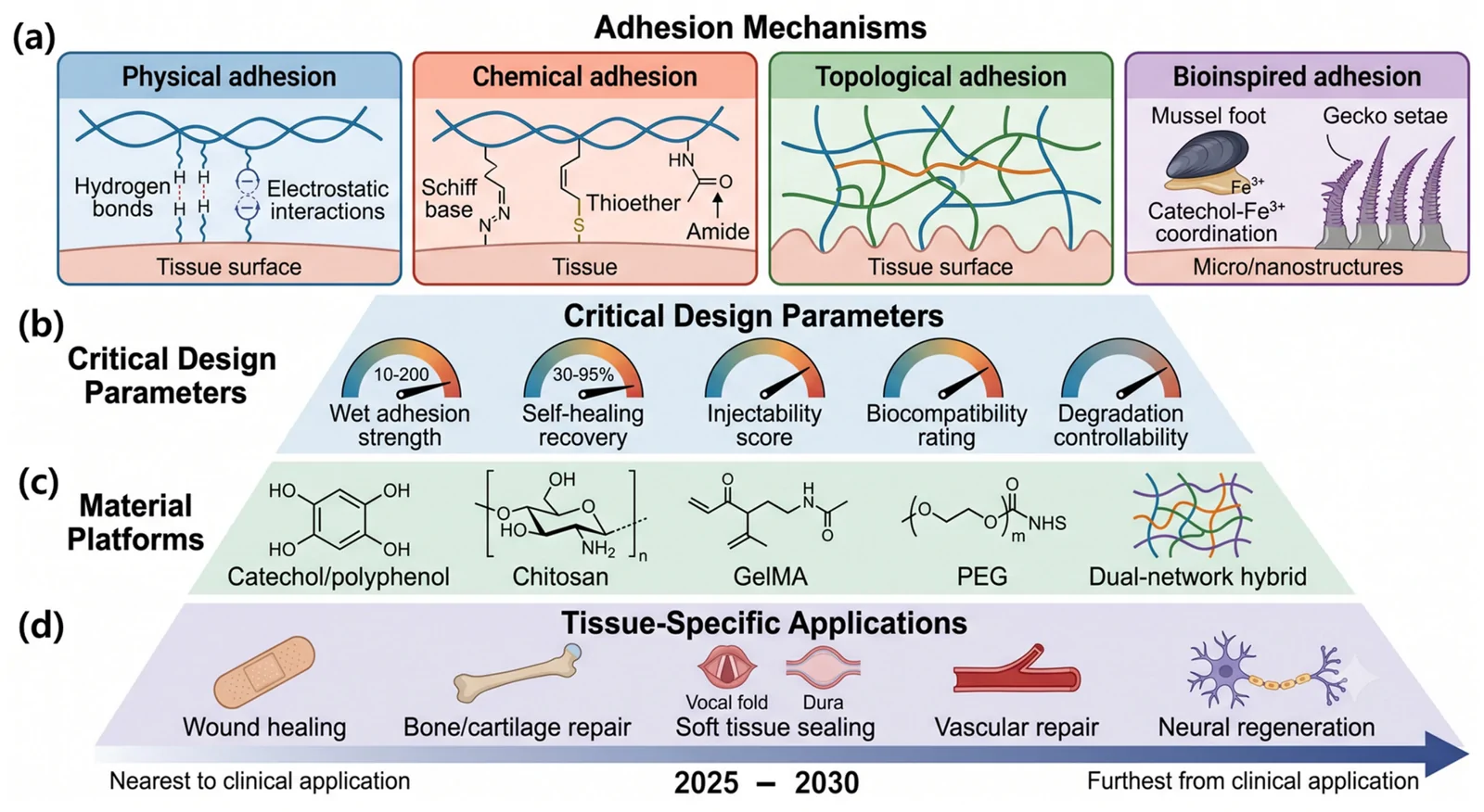
Overview of bioadhesive hydrogel design and application hierarchy: (**a**) adhesion mechanisms (physical, chemical, topological, bioinspired); (**b**) critical design parameters (adhesion strength, self-healing, injectability, biodegradability); (**c**) material platforms (catechol/polyphenol, chitosan, GelMA, PEG, dual-network hybrids); and (**d**) tissue-specific applications with translational timelines.

Understanding the fundamental mechanisms governing hydrogel-tissue adhesion is essential for rational design of high-performance bioadhesives. Four principal adhesion mechanisms have been identified, each offering distinct advantages and limitations that inform material selection and optimization strategies (Figure 1a). In practice, the most effective bioadhesive systems combine multiple mechanisms synergistically to achieve robust, reliable adhesion under challenging physiological conditions.

### 2.1. Physical Adhesion

Physical adhesion mechanisms operate through non-covalent interactions including hydrogen bonding, electrostatic interactions, van der Waals forces, and hydrophobic interactions (Figure 1a). These weak interactions offer distinct advantages, including reversible and non-destructive adhesive release, mild processing conditions compatible with biomolecules, and straightforward tunability through compositional changes [15].

**Figure 2:**
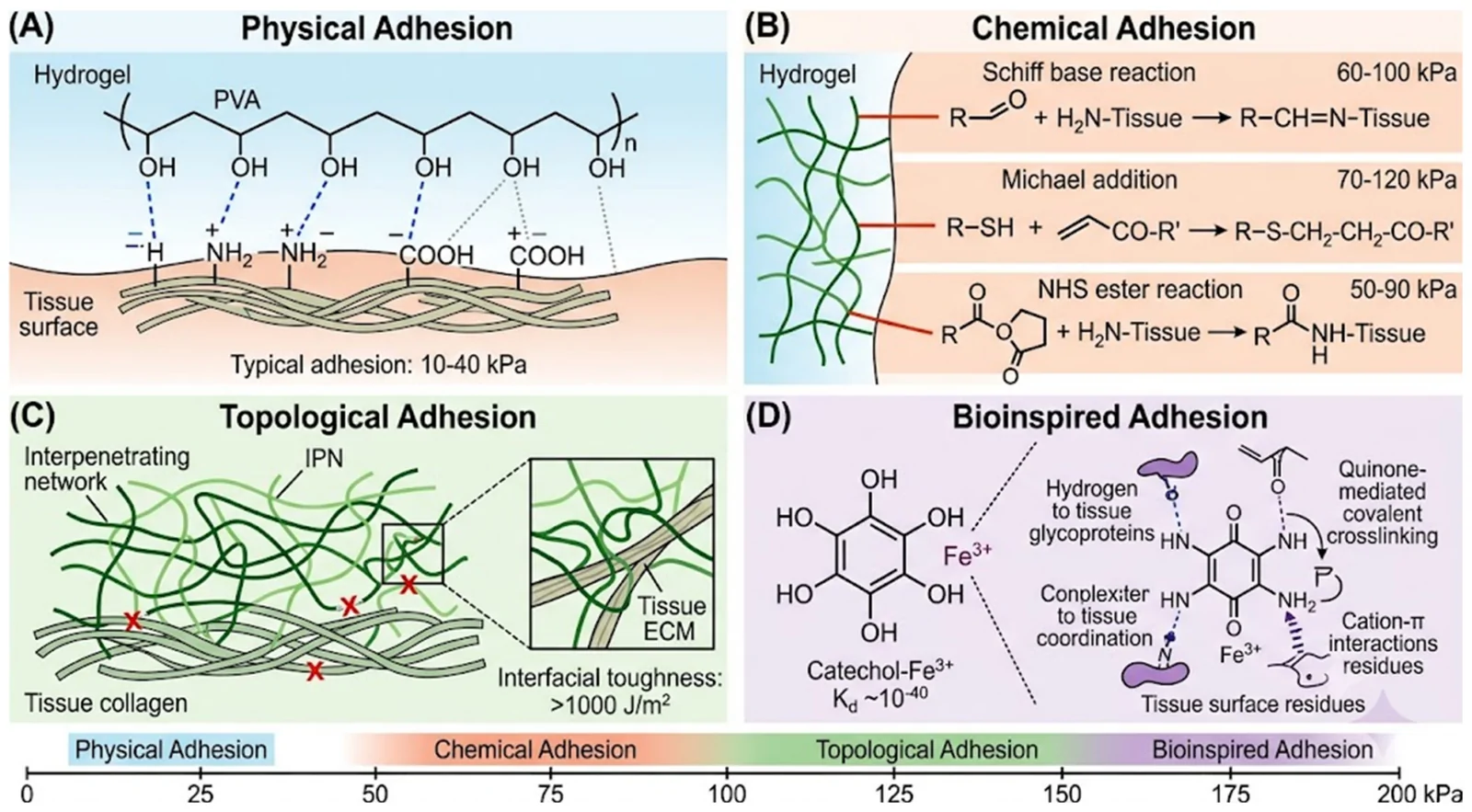
Molecular mechanisms of adhesion with quantitative ranges: (**A**) physical adhesion through hydrogen bonding and electrostatic interactions (typical adhesion: 10–40 kPa); (**B**) chemical adhesion via covalent crosslinking—Schiff base (60–100 kPa), Michael addition (70–120 kPa), NHS ester (50–90 kPa); (**C**) topological adhesion from interpenetrating networks (100–180 kPa); and (**D**) bioinspired adhesion from catechol–metal coordination (80–150 kPa) and van der Waals forces (50–100 kPa). Each mechanism includes representative chemical structures and quantitative adhesion strength ranges from lap-shear testing.

Polyvinyl alcohol (PVA)-based hydrogels achieve adhesion primarily through extensive hydrogen bonding networks with tissue proteins and extracellular matrix components. The hydroxyl groups on PVA chains form multiple hydrogen bonds with amide, carboxyl, and hydroxyl groups present on tissue surfaces, creating a reversible adhesive interface. Similarly, polyelectrolyte complex hydrogels formed from oppositely charged polysaccharides such as sodium alginate and chitosan adhere to tissues through electrostatic interactions, stabilizing the polymer-tissue interface [16] (Figure 1c and Figure 2A).

The strength of physical adhesion is governed by the density of non-covalent interactions per unit area and the thermodynamics of bond formation/dissociation. Individual hydrogen bonds contribute approximately 5–30 kJ/mol, while electrostatic ion pairs provide 5–20 kJ/mol, and van der Waals interactions contribute only 0.4–4 kJ/mol [15]. Although these interactions are individually weak, their cumulative effect across millions of binding sites per square centimeter can generate substantial macroscopic adhesion.

Supramolecular strategies can further enhance physical adhesion while preserving reversibility. Host–guest interactions such as cyclodextrin-adamantane and cucurbituril–ferrocene complexes provide relatively strong non-covalent binding with individual binding energies of 20–100 kJ/mol [17].

However, physical adhesion mechanisms suffer critical limitations in physiological wet environments. The high water content of biological tissues competes for hydrogen bonding and disrupts electrostatic interactions, dramatically reducing adhesion strength. Typical physical adhesives demonstrate shear strengths of only 5–20 kPa on wet tissue surfaces, inadequate for most surgical applications [18]. Furthermore, the reversibility that enables benign release limits load-bearing capacity during healing phases. Zwitterionic hydrogels represent an interesting middle ground, where balanced positive and negative charges create strong hydration layers while maintaining tissue-interactive capacity. Pei et al. demonstrated zwitterionic hydrogels achieving tissue adhesion through charge-driven interactions while providing strain-sensing capability for organ motion monitoring [18]. Consequently, physical adhesion mechanisms serve primarily as secondary reinforcement in hybrid systems or for applications requiring temporary, removable adhesion such as wearable biosensors and drug delivery patches (Figure 1a).

### 2.2. Chemical Adhesion

Chemical adhesion relies on covalent bond formation between hydrogel and tissue, creating durable mechanical interfaces resistant to aqueous disruption. Primary strategies dominate the field: Schiff base chemistry, Michael addition and NHS (N-hydroxysuccinimide) ester crosslinking. Each differs in kinetics, reversibility, and biocompatibility profiles [19,20] (Figure 2B).

Schiff base formation between aldehyde groups on the hydrogel and primary amines on tissue proteins (predominantly lysine residues, constituting approximately 5–7% of amino acid residues in collagen) creates reversible imine bonds stabilized by resonance effects. This approach offers advantages of rapid gelation (minutes to hours), tunable crosslinking density, and partial reversibility under acidic conditions.

The aldehyde component is most commonly supplied by oxidized polysaccharides. Oxidized dextran, oxidized hyaluronic acid, and oxidized alginate react with tissue amines and with amino-functionalized polymer chains simultaneously to achieve interfacial bonding and bulk network formation. Ren et al. developed injectable hydrogel adhesives using catalyst-free o-phthalaldehyde/amine crosslinking, achieving rapid, firm adhesion to a range of tissues and effective sealing of liver and blood-vessel incisions [20].

The principal limitation of this chemistry is not how fast imine bonds form but how few of them persist. Imine condensation is a reversible, thermodynamically controlled equilibrium in which water is simultaneously the leaving group and the bulk solvent, so an aqueous environment intrinsically favours the hydrolysed state, and the crosslink population is continuously renewed rather than fixed [21]. In an implanted adhesive the equilibrium is additionally open: interstitial flow and lymphatic clearance remove the liberated aldehyde and amine from the interface, displacing the equilibrium further toward dissociation. The result is a gradual decline in interfacial crosslink density rather than a discrete failure event.

Hydrolysis is not the only depletion pathway. Imines also undergo associative exchange—transimination with free amines and imine metathesis—without any net hydrolysis occurring [21]. The physiological milieu supplies a large, continuously replenished pool of competing primary amines. Consistent with this, Schiff-base hydrogels respond not only to pH but to free amino acids and other amine-bearing metabolites, precisely because the linkage remains in equilibrium with its aldehyde and amine reactants [22]. Dynamic hyaluronic acid networks crosslinked through imine and hydrazone linkages lose structural integrity within roughly one to three days of aqueous incubation, whereas isostructural oxime networks persist substantially longer [23].

By contrast, Schiff bases exhibit kinetic instability under physiological pH (half-life of hours to days at pH 7.4), gradually hydrolyzing and reducing long-term adhesive strength. Aromatic Schiff bases are formed between aromatic aldehydes and amines. Extended conjugation makes them more stable than aliphatic analogs, which offers one route to improved longevity [24].

Michael addition adds thiols or amines to electron-deficient double bonds such as acrylates, maleimides, and vinyl sulfones. The resulting thioether or secondary amine bonds are irreversible and far more stable than Schiff bases under physiological conditions. This mechanism enables gelation through mixing complementary components without external triggers, facilitating clinical application.

Kinetic tunability constitutes the principal design lever of this chemistry. The reaction kinetics can be tuned from seconds to hours by adjusting nucleophile/electrophile pair selection and concentration. Thiol–maleimide pairs react rapidly (seconds) at physiological pH, while thiol–acrylate pairs require minutes to hours, providing broader working time windows for surgical application [25]. Multi-arm PEG-based systems with thiol and maleimide functionalities achieve adhesion strengths of 30–80 kPa on wet tissue surfaces through simultaneous tissue-reactive crosslinking and bulk network formation.

NHS ester chemistry, commonly employed in PEG-based adhesives (e.g., CoSeal), rapidly reacts with primary amines to form stable amide bonds. NHS esters are stable in storage and generate strong adhesion. They also hydrolyze rapidly in water, with a half-life of about 10 min at pH 7.4 and 37 °C, so they must be handled carefully [3]. CoSeal, composed of 4-arm PEG-NHS and 4-arm PEG-NH2, seals blood vessels within 1 min with hemostatic efficacy of 86% in clinical application. DuraSeal is based on tetra-PEG-succinimidyl ester and trilysine amine. It reaches a higher efficacy of 98.2%, but it swells by 50~400% after application. This swelling can compress the spinal cord and nerves [25]. This illustrates a critical limitation of PEG-NHS systems: uncontrolled swelling in confined anatomical spaces.

A significant limitation of all chemical adhesion strategies involves potential cytotoxicity from unreacted functional groups, byproducts, or formaldehyde generation from Schiff base hydrolysis. This necessitates careful optimization to balance adhesion strength against cell viability. Recent advances in bioorthogonal chemistry, click reactions proceeding under physiological conditions without side reactions with biological molecules, offer promising solutions. Copper-free strain-promoted azide-alkyne cycloaddition (SPAAC) and inverse electron-demand Diels–Alder reactions between tetrazine and norbornene enable rapid, selective crosslinking without catalysts or cytotoxic intermediates [26]. Takahashi et al. demonstrated in situ crosslinkable hyaluronan hydrogels via copper-free click chemistry, achieving rapid gelation with excellent biocompatibility [27].

### 2.3. Topological Adhesion

Topological adhesion operates through mechanical interlocking at the hydrogel-tissue interface, wherein the hydrogel network physically entangles with tissue surface roughness and fibrous extracellular matrix components. This mechanism does not require covalent bonds or specific chemical interactions; rather, adhesion emerges from geometric complementarity and energy barriers to delamination [28]. The dry double-sided tape concept pioneered by Yuk et al. exemplifies topological adhesion through a three-layer architecture: a water-repelling outer layer minimizing interfacial water, an absorptive inner layer rapidly removing water from the tissue surface, and mechanical interlocking throughout the adhesive zone [7]. This approach achieved interfacial toughness exceeding 1000 J/m^2^ on wet porcine skin, rivaling the strongest cyanoacrylate adhesives (Figure 2C).

The mechanism can be understood through the Griffith energy balance framework: delamination requires simultaneous rupture of multiple entanglement points, with each point contributing an energy barrier proportional to the chain length between entanglements and the number of physical crosslinks in the entanglement zone [29]. The bulk hydrogel dissipates further energy during delamination. Hysteretic processes such as chain scission and sacrificial bond breakage raise the apparent adhesion energy well above the intrinsic interfacial bond energy. This toughening mechanism is analogous to the large-scale energy dissipation observed in pressure-sensitive adhesives and biological adhesion systems.

Topological adhesion provides distinct advantages, including independence from chemical functionality (broadening applicability to diverse tissue types), reversibility upon mechanical peeling, and reduced cytotoxicity from eliminated chemical crosslinkers. Yet, adhesion strength depends critically on tissue surface properties, roughness, and collagen density, creating significant variability between tissue types and processing conditions. Furthermore, topological adhesion exhibits directional dependence; shear adhesion often exceeds peel strength by 3–10 fold due to differences in stress distribution during delamination [30]. Interpenetrating network (IPN) hydrogels enhance topological adhesion by expanding the physical entanglement zone, increasing the number of contact points between the hydrogel and tissue. Fang et al. developed fibrillar connected double-network hydrogels with a toughness of 55 MJ/m^3^. A PAAm network was chemically crosslinked by an acrylated agarose fibrillar network. Hierarchical architecture therefore has a large effect on mechanical performance [30].

Recent advances in 3D-printed adhesive hydrogels leverage topological adhesion by creating precisely designed surface microstructures that maximize mechanical interlocking with tissue surfaces. Wu et al. developed biomimetic 3D-printed adaptive hydrogel bioadhesives featuring octopus-inspired suction cup microstructures combined with catechol chemistry, achieving superior adhesion and infection resistance in challenging wet tissue environments [31]. This represents a convergence of topological and chemical adhesion mechanisms enabled by advanced manufacturing.

### 2.4. Bioinspired Adhesion

Nature has evolved sophisticated adhesion mechanisms over millions of years, refined for performance in wet, dynamic environments. Bioinspired adhesion strategies translate these principles into synthetic materials, offering design paradigms that have driven many of the most significant advances in bioadhesive hydrogels [32,33] (Figure 2D). Each biological model operates under conditions a surgical site does not reproduce-a controlled a secretory microenvironment, an inert mineral or shell substrate, permanent rather than resorbable attachment, and no requirement for host compatibility. The following analysis therefore evaluates each strategy not by the adhesion values reported but by whether its underlying mechanism survives transfer to physiological conditions.

Mussel-inspired adhesion, derived from marine mussel byssus proteins, centers on catechol (3,4-dihydroxyphenyl) chemistry. Mussels accumulate the amino acid L-DOPA (dihydroxyphenylalanine) in adhesive plaque proteins, chiefly Mfp-3 and Mfp-5, at concentrations up to 30 mol%. Its catechol groups adhere through four concurrent mechanisms. They hydrogen-bond with tissue glycoproteins, contributing roughly 2–5 kPa per catechol. Quinones crosslink with lysine, cysteine and histidine. Catechols coordinate Fe^3+^ and other multivalent cations. They also form cation-π interactions with positively charged tissue residues [34,35]. Lee et al. first demonstrated that synthetic dopamine-functionalized polymers recapitulate mussel adhesion in 2007, initiating a research explosion in catechol-based materials [34]. Waite’s analysis of mussel adhesion showed that the spatial arrangement of DOPA residues matters. They concentrate at the plaque-substrate interface, and this arrangement guides the design of synthetic catechol-presenting materials [36].

Catechol chemistry offers remarkable advantages: multiple adhesion pathways provide redundancy and robustness; pH-dependent quinone formation enables stimuli-responsive behavior; Fe^3+^ complexation creates reversible yet strong crosslinks ideal for self-healing (mono-, bis-, and tris-catechol-Fe^3+^ complexes with stability constants of 10^24^, 10^37^, and 10^40^, respectively); and catechol is naturally derived with inherent biocompatibility [37] (Figure 1c). Dopamine-functionalized PEG, hyaluronic acid, and chitosan have achieved wet adhesion strengths of 50–150 kPa, approaching or exceeding commercial benchmarks (Figure 1b,c). Shin et al. developed catechol-modified hyaluronic acid hydrogels achieving effective, minimally invasive cell therapy through tissue-adhesive properties [35]. Recent innovations have extended catechol chemistry to achieve unprecedented adhesion. Chen et al. synthesized catechol derivatives carrying long aliphatic side chains of about 10 atoms. The resulting hydrogels reached an adhesive tensile strength near 1800 kPa and an interfacial toughness on wet porcine skin near 1300 J/m^2^, with on-demand detachment [38].

Tannic acid (TA), a plant-derived polyphenol containing 10–40 phenolic hydroxyl groups per molecule, has emerged as a particularly promising and cost-effective alternative to dopamine. Synthesized catechol derivatives carry long aliphatic side chains of about 10 atoms. The resulting hydrogels reached an adhesive tensile strength near 1800 kPa and an interfacial toughness on wet porcine skin near 1300 J/m^2^, with on-demand detachment [39,40]. Wet adhesion of TA-based adhesives (50–120 kPa) is comparable to that of dopamine systems. TA is also about 100-fold cheaper than synthetic dopamine derivatives, simpler to process, and already established as safe in food and pharmaceutical use. Recent work established TA as a multifunctional bioactive polyphenol for wound healing hydrogels. A single material can combine adhesion with antibacterial, antioxidant, anti-inflammatory and hemostatic effects [41]. Kim et al. developed TAPE (TA-inspired PEG), a medical adhesive demonstrating that ubiquitous plant compounds can rival specialized mussel-inspired chemistry [42].

Critically, catechol-based systems exhibit critical limitations. Catechol oxidation to quinone species, an essential step for crosslinking, generates reactive oxygen species and reduces adhesivity if excessive. This necessitates antioxidant incorporation (ascorbic acid, sodium sulfite) or oxygen-free processing [43,44]; because that oxidative burden falls on adjacent cells, reported cytocompatibility is formulation-specific and does not generalize across catechol content or curing conditions. Fe^3+^ coordination is subject to a separate limitation. Catecholato- Fe^3+^ speciation is pH-dependent, with mono-complexes predominating below pH 5, bis-complexes near pH 8, and tris-complexes only under alkaline conditions [44], so the tris-complexes stability constant commonly cited as design justification is not the species that dominates at physiological pH. Free Fe^3+^ is in any case scarce in vivo owing to limited availability in physiological conditions due to high-affinity iron-binding proteins (transferrin, lactoferrin), potentially reducing in vivo adhesion compared to in vitro performance.

Gecko-inspired dry adhesion mechanisms exploit hierarchical micro/nanostructures generating high contact area with tissue surfaces through van der Waals interactions. Autumn et al. provided evidence that gecko setae achieve adhesion primarily through van der Waals forces rather than chemical bonding, with individual setal adhesion forces of approximately 200 μN [40]. While gecko setae excel in dry environments, their application to wet tissue remains challenging due to water disruption of van der Waals contacts. Hybrid approaches combining gecko-inspired topography with catechol chemistry or chemical crosslinking show promise for wet tissue adhesion.

Sandcastle-worm- and barnacle-inspired adhesion, based on protein-rich underwater adhesives that cure rapidly in seawater, emphasizes protein denaturation and polyphenol chemistry. Stewart et al. revealed that natural underwater adhesives from sandcastle worms exploit complex coacervation of proteins with polyanions, demonstrating that phase-separated microdomains can generate strong, tough interfaces [45]. This principle has been adapted to synthetic hydrogels through polyelectrolyte complexation, creating adhesives that function effectively in aqueous environments.

### 2.5. Comparative Assessment of Adhesion Mechanisms

The four adhesion mechanisms present distinct advantages and trade-offs. Physical adhesion (hydrogen bonding, electrostatic interactions) achieves modest adhesion strength (10–40 kPa) but offers simplicity, rapid formation without chemical reactions, and inherent reversibility—valuable for temporary wound dressings but insufficient for structural tissue repair (Figure 2A). Chemical adhesion via covalent crosslinking dramatically increases wet adhesion strength (Schiff base: 60–100 kPa; Michael addition: 70–120 kPa; NHS ester: 50–90 kPa) but sacrifices reversibility and requires complex multi-component formulations or UV/thermal activation (Figure 2B). Topological adhesion through interpenetrating networks achieves excellent adhesion (100–180 kPa) combined with superior self-healing (75–95% recovery) by distributing stress through redundant mechanical pathways, though synthesis is more complex and gelation times are longer (Figure 2C).

Bioinspired catechol–metal coordination represents an optimal middle ground: achieving high wet adhesion (80–150 kPa), tunable self-healing (60–90%), and compatibility with diverse polymer backbones and chemical modifications. Catechol adhesion is pH-sensitive, peaking near pH 8–9 and declining below pH 6 or above pH 10. This is an advantage because it confers environmental responsiveness. It is also a limitation, because performance varies in diseased or acidic wounds (Figure 2D). Current evidence favours combination strategies. Integrating catechol coordination with topological contributions in dual networks gives the most clinically promising platforms. Chemical bonding supplies high initial adhesion, and mechanical interlocking supplies long-term durability. Such hybrid mechanisms are now the focus of 60+ recent publications and represent the consensus direction for next-generation bioadhesives [12,14,19,46,47].

A critical gap persists. Most reported adhesion values come from idealized laboratory conditions, with clean tissue surfaces, controlled temperature and standardized substrates. These conditions poorly represent bleeding, inflamed or infected wound beds. In our own work on vocal fold bioadhesives, the gap between in vitro and in vivo adhesion often exceeded 50%. Blood, mucus and dynamic tissue motion are the main causes, and no bench-top test reproduces them adequately [14]. This reproducibility gap remains the single most underappreciated barrier to clinical translation, and the field urgently needs standardized wet adhesion protocols that incorporate physiologically relevant confounders.

## 3. Key Design Parameters

The translation of adhesion mechanisms into clinically useful bioadhesive hydrogels requires careful optimization of multiple interdependent design parameters. These parameters must be balanced against each other and tailored to specific tissue and application requirements, creating a multidimensional optimization challenge that defines modern bioadhesive engineering.

### 3.1. Adhesion Strength vs. Biocompatibility

A fundamental design tension exists between adhesive strength and tissue biocompatibility. Strong chemical crosslinkers often require toxic catalysts, generate hazardous byproducts, or inhibit cell adhesion and proliferation. The ideal bioadhesive achieves sufficient bonding strength for the intended application while maintaining cytocompatibility, enabling cell migration, differentiation, and tissue remodeling [48]. Adhesion strength is quantified through standardized tests: lap shear testing measures shear strength in N/cm^2^; tensile testing directly applies peel forces; burst pressure testing evaluates holistic integrity under pressure; and interfacial toughness (J/m^2^) provides fracture-mechanics-based characterization [14].

Strength requirements vary widely by application. Skin wound closure requires 10–30 kPa shear strength, and bone adhesion typically requires 50–200 kPa. Vascular sealants must tolerate burst pressures of 100–300 mmHg, or 13–40 kPa. Cardiac patches need both strength and elasticity to withstand systolic/diastolic cycling of roughly 10–20% strain at 1–2 Hz. Neural applications instead demand very soft moduli of 0.1–1 kPa to match brain tissue [44]. Commercial products provide reference points. Fibrin glue achieves about 10 kPa in wet environments. Cyanoacrylate exceeds 100 kPa but fails in a brittle manner. CoSeal provides 15–25 kPa. Leading experimental hydrogels reach 100–200 kPa while remaining elastic [6,7].

The apparent paradox that stronger adhesives often exhibit reduced biocompatibility reflects the chemical reactivity required for strong covalent crosslinking. Highly reactive functional groups (aldehydes, NHS esters, isocyanates) that form strong tissue bonds also react with cellular proteins, disrupting membrane integrity and intracellular signaling. Dynamic covalent bonds offer a way around this paradox. Disulfide exchange gives strong bonds that break and reform under reducing conditions. Boronate esters give glucose-responsive reversible bonds. Schiff bases give pH-sensitive reversibility. These dynamic bonds enable strong adhesion during the critical early healing period before gradually releasing as tissue remodeling progresses, avoiding chronic inflammatory responses associated with permanent chemical modification.

It is worth noting, however, that the field’s emphasis on maximizing adhesion strength may itself be misguided. Clinical success does not necessarily correlate with the highest adhesion values; rather, matching adhesion to tissue-specific mechanical requirements without compromising robust biocompatibility is the more relevant engineering objective. Many studies report impressive adhesion numbers (>200 kPa) achieved through aggressive chemical crosslinking, yet these same systems often show reduced cell viability below 70%, well below the ISO 10993-5 threshold for clinical acceptability. A paradigm shift toward “sufficient adhesion with optimal biocompatibility” rather than “maximum adhesion” would better serve clinical translation goals (Table 1).

### 3.2. Self-Healing Capability

Self-healing hydrogels autonomously repair mechanical damage through dynamic bond breaking and reformation, prolonging adhesive longevity under cyclic loading. Biological tissues constantly experience mechanical deformation, skin stretches up to 75% during joint movement, cardiac tissue undergoes 10–20% cyclic strain at 1–2 Hz, and vocal folds vibrate at 100–300 Hz during phonation, requiring adhesive interfaces that accommodate repeated stress without cumulative damage [49,50].

Multiple dynamic bond types enable self-healing, with characteristic healing kinetics and strength recovery profiles. Hydrogen bonds (weak, ~5–30 kJ/mol) heal within seconds but provide limited strength recovery (30–60%). Metal–ligand coordination complexes, particularly Fe^3+^-catechol (per bond ~100 kJ/mol) and Cu^2+^-histidine, offer rapid association/dissociation kinetics with good strength recovery (70–90% within 24 h) [50]. Diels-Alder reversible cycloadditions provide thermally responsive healing but require elevated temperatures (60–80 °C), limiting physiological applicability. Boronate esters respond to diol concentration and pH, creating glucose-responsive self-healing suitable for diabetic wound applications. Disulfide exchange, activated by reducing conditions (glutathione, cysteine), enables intracellular or wound-microenvironment-triggered healing [51].

Fe^3+^-catechol coordination represents the most extensively studied self-healing strategy, combining rapid kinetics with reasonable bond strength. Experimental hydrogels incorporating Fe^3+^-catechol demonstrate 70–90% strength recovery within 24 h following mechanical damage, with some advanced systems achieving >95% recovery within 3 min [52]. Engineering self-healing therefore requires a balance. Bond exchange that is too rapid compromises mechanical stability under continuous load, causing creep. Healing that is too slow leaves inadequate repair between loading cycles.

Recent advances employ multiple dynamic bond types simultaneously (dual-dynamic or triple-dynamic networks) to independently optimize strength and healability. For example, combining fast-healing hydrogen bonds for rapid initial recovery with slower-healing metal coordination for long-term strength restoration creates hierarchical self-healing behavior. Metal coordination systems raise two further issues: physiological ion concentrations and metal ion toxicity. Fe^3+^ above 50 μM can drive Fenton-mediated oxidative stress, so biocompatibility must be validated carefully [53].

### 3.3. Wet Adhesion

Adhesion in physiological wet conditions represents the preeminent challenge in bioadhesive design. Water molecules at interfaces competitively interact with both the adhesive and tissue, disrupting hydrogen bonds, shielding electrostatic interactions, and diluting reactive chemical groups. This fundamentally explains why traditional synthetic adhesives (cyanoacrylates, epoxies) fail in wet environments: their adhesion mechanisms depend on water-free interfaces [54].

Three principal strategies address wet adhesion. First, water-repelling interfacial layers mitigate water infiltration at the adhesion zone. Hydrophobic polymers, fluorinated compounds and lipophilic modifications all create interfacial barriers. The dry double-sided tape achieves the same effect with a dry PAA-NHS ester layer. This layer rapidly absorbs interfacial water and creates a transiently dry interface, so strong covalent bonds can form [7]. Shen et al. (2025) developed wet-adhesion and swelling-resistant hydrogel bioadhesives through balanced hydrophilic-hydrophobic network design, achieving robust adhesion while preventing the post-application swelling that plagues PEG-based systems [51].

Second, reactive chemistry addressing water competition involves rapid covalent bond formation outpacing water diffusion. High-valency crosslinkers saturating reactive groups before hydrolysis, for example, multi-arm PEG-NHS esters reacting with tissue amines within seconds, create a kinetic advantage. Catechol chemistry exploits redox-catalyzed oxidation to quinone-imine species that immediately crosslink with tissue amines, effectively sealing the interface against water penetration [55]. Xu et al. demonstrated pH-independent and ultrafast gelation bioadhesive hydrogels achieving gastric ulcer healing in porcine models, where rapid gelation (seconds) in the highly acidic gastric environment represented extreme wet adhesion [53].

**Figure 3:**
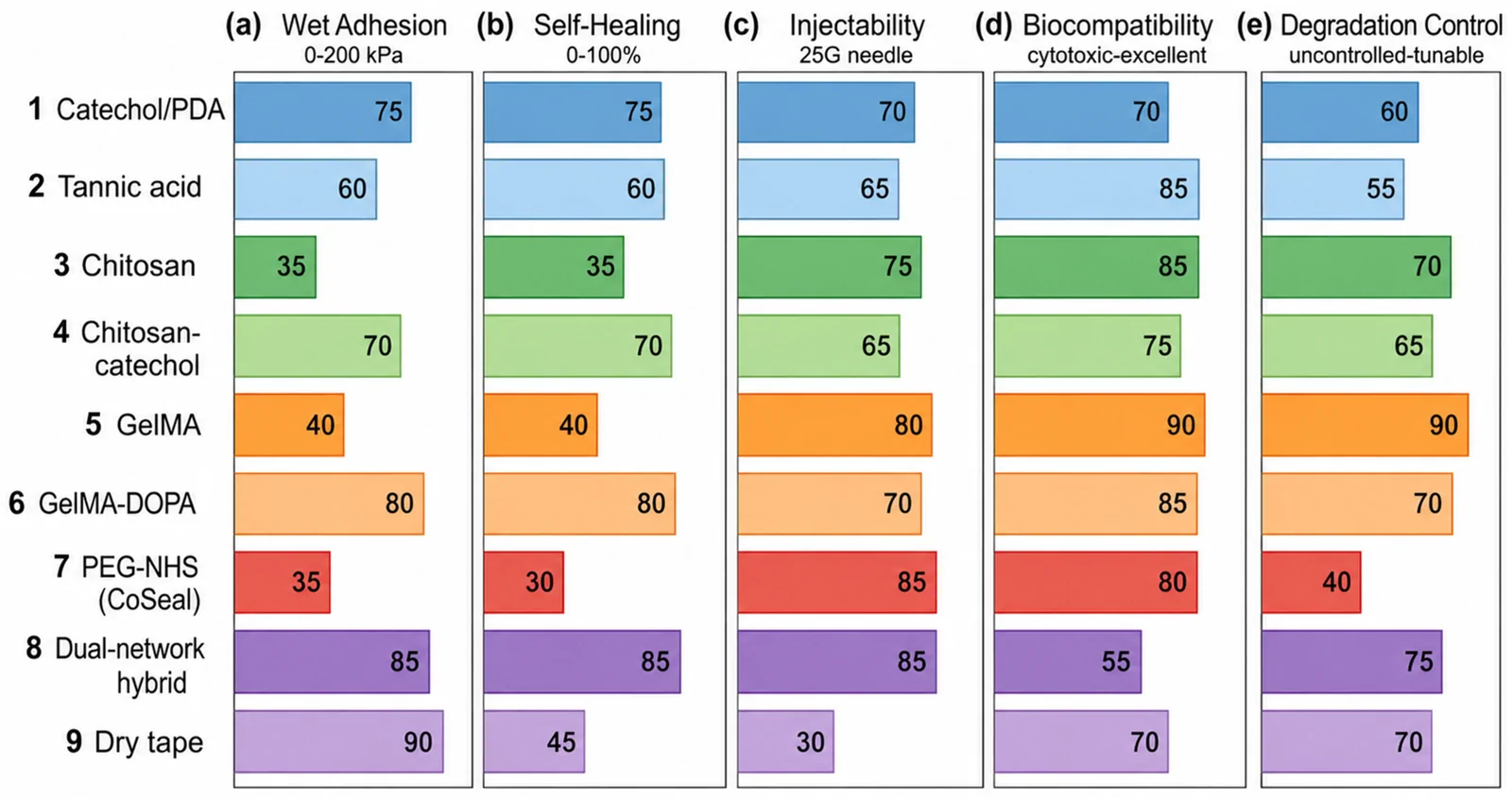
Design parameter trade-off analysis: (**a**) wet adhesion strength; (**b**) self-healing recovery %; (**c**) injectability score; (**d**) biocompatibility rating; and (**e**) degradation controllability. Radial axis scale normalized 0–100.

Third, topological interlocking provides a water-independent strategy. Sufficient mechanical entanglement with tissue surface roughness and ECM fibrils creates energy barriers to delamination that persist in aqueous environments. This mechanism underlies the effectiveness of the dry double-sided tape approach, where rapid water absorption creates space for polymer chain interpenetration with tissue surfaces (Figure 3a).

Quantitative wet adhesion benchmarking remains inconsistent across the literature, differing tissue types (fresh vs. fixed, species variation), surface preparation methods (blotting vs. immersion), and test protocols (lap shear vs. peel vs. tensile) generate 5–10-fold variation in reported values for identical adhesives [14]. The absence of standardized wet adhesion testing protocols remains a significant barrier to meaningful cross-study comparison and clinical translation planning.

### 3.4. Injectability and In Situ Gelation

Minimally invasive surgical techniques demand injectability through standard syringes and catheters (18–25 gauge, corresponding to inner diameters of 0.84–0.26 mm) with rapid in situ gelation at the target tissue site [56]. Injectability requires shear-thinning behavior wherein high shear rates during injection (10^2^–10^4^ s^−1^) reduce viscosity, enabling passage through narrow lumens, followed by viscosity rapid recovery post-injection preventing gel leakage before complete gelation [57].

Gelation Triggers Can Be Classified by Mechanism:

Temperature-responsive systems gel at body temperature without any external trigger, which makes them simple to use. Poly(N-isopropylacrylamide) (PNIPAM) has a lower critical solution temperature near 32 °C. Pluronic polymers gel above their critical micelle concentration. These systems have four drawbacks. Gelation is often incomplete. Syneresis expels 10–30% of the volume as water during aging. Gelation rate is difficult to control. PNIPAM can also be cytotoxic at higher concentrations [58]. A recent thermo-switchable underwater adhesive based on a Janus hydrogel modulated adhesion reversibly with temperature. Adhesion strength differed by more than 1000-fold between the adhesive and non-adhesive states [59].

Photocrosslinking through methacrylate-functionalized polymers (GelMA, HAMA) enables precise spatial and temporal control via focused visible or UV light, ideal for surgical visualization and localized sealing. Recent advances in visible-light photoinitiator systems (eosin Y, ruthenium complexes, lithium phenyl-2,4,6-trimethylbenzoylphosphinate/LAP) reduce phototoxicity compared to traditional UV-activated Irgacure 2959 [60]. However, photocrosslinking depth is limited by light penetration (typically <2 mm in tissue for UV, up to 5–10 mm for visible/NIR light), and retained photoinitiator residues generate ROS toxicity concerns above approximately 0.05% *w*/*v* [61].

Enzymatic gelation is elegant because it starts automatically on contact with tissue. Endogenous enzymes act as the trigger. Examples include transglutaminase for glutamine-lysine crosslinks, horseradish peroxidase with hydrogen peroxide, and tyrosinase for DOPA crosslinking. Enzyme activity varies between patients, tissue types, and disease states, creating unpredictable gelation kinetics [62].

Chemical mixing-based systems (two-component adhesives) provide the most predictable, rapid gelation through stoichiometric control but require specialized dual-barrel delivery devices and pose application complexity in clinical settings. Innovations in microfluidic mixing and spray-based delivery systems address these practical limitations [63] (Figure 3c).

### 3.5. Biodegradability and Degradation Control

The required lifetime depends on the application. Temporary hemostatic dressings should degrade within days to weeks. Tissue scaffolds should persist for months to match healing timelines. Some structural repairs may warrant near-permanent materials [64]. Two degradation mechanisms operate. Hydrolytic cleavage of backbone and ester linkages follows pseudo-first-order kinetics that depend on water content, pH and temperature. Enzymatic degradation acts through matrix metalloproteinases (MMP-1, MMP-2, MMP-9), collagenase, hyaluronidase and serine proteases, which cleave specific peptide sequences [65].

Enzymatic degradation provides the advantage of concentration-dependent kinetics; degradation rate increases proportionally to local protease activity, theoretically synchronizing adhesive dissolution with wound healing progression. MMP-cleavable peptide sequences (GPQG↓IWGQ and variants) incorporated into crosslinkers create “cell-demanded” degradation where migrating cells locally degrade the adhesive as they remodel tissue [66]. Complicating this approach, MMP activity varies dramatically between individuals, wound types (3–100-fold variation between chronic and acute wounds), and disease states (elevated in diabetic wounds), introducing unpredictable degradation behavior.

Degradation byproducts represent critical biocompatibility considerations. Acidic degradation products from polylactic acid and polyglycolic acid components accumulate, lowering local pH by 0.5–2.0 units and potentially triggering adverse inflammatory responses. Oligomeric fragments below the renal filtration threshold (~5–10 kDa) can accumulate in off-target tissues, particularly liver and kidney. Ren et al. achieved tunable in vivo degradation of 6 to 22 weeks by incorporating disulfide bonds into the same o-phthalaldehyde-crosslinked network, enabling resorption matched to the healing timeline [20]. Tunable degradation via crosslink density, monomer composition, and peptide sequence selection enables matching degradation rate to tissue healing timeline, typically 2–12 weeks for dermal wounds, 6–24 weeks for bone defects, and 4–12 weeks for soft tissue repair (Figure 3e).

## 4. Material Platforms

The diversity of adhesion mechanisms and design parameters has driven development of multiple material platforms, each with characteristic strengths, limitations, and optimal application domains. This section systematically evaluates the major material classes with emphasis on recent advances (2020–2025) and quantitative performance benchmarking.

### 4.1. Catechol/Polyphenol-Based Systems

Catechol-functionalized polymers represent the most extensively validated bioadhesive platform, building upon decades of mussel biology research. This maturity, however, reflects breath of publication more than depth of validation: the adhesion values reported across the subcategories below are obtained under heterogeneous test geometries (lap shear, burst pressure), substrates and hydration states, and are therefore only semi-quantitatively comparable [67]. The platform has diversified into several mature subcategories [32,68]:

Dopamine (3,4-dihydroxyphenethylamine) conjugation to polymer backbones through EDC/NHS coupling or other amide-forming chemistry creates catechol-presenting materials. HA-DOPA (hyaluronic acid-dopamine) achieves wet adhesion of 50–100 kPa while maintaining excellent biocompatibility and hyaluronidase-mediated biodegradability [41]. PEG-DOPA systems offer precise molecular weight control (3–40 kDa) and tunable crosslink density; the trade-off is that the same inert architecture that permits this control provides no intrinsic bioactivity and a non-degradable backbone, so failure in these systems is typically cohesive rather than interfacial. Chitosan–dopamine conjugates combine catechol adhesion with inherent antimicrobial activity and pH-responsive gelation [68]; here the practical cost is a pH conflict, in that chitosan requires acidic dissolution, whereas catechol oxidative coupling proceeds efficiently only at neutral-to-alkaline pH, coupling gelation kinetics and adhesion strength to a narrow operating window that is difficult to maintain across different tissue sites [69]. Ryu et al. demonstrated catechol-functionalized chitosan/pluronic hydrogels achieving simultaneous tissue adhesion and hemostatic activity through cooperative catechol–chitosan interactions [61]. The catechol conjugation degree (typically 5–30 mol%) critically influences adhesion; higher substitution increases adhesion but reduces polymer solubility and increases quinone-mediated cytotoxicity.

This trade-off is, however, more tractable than it is usually presented. Guyot et al. showed that the cytotoxicity of catechol–chitosan hydrogels tracks the leaching of free quinone rather than H_2_O_2_ generation or catechol content per se, and that increasing the degree of oxidative crosslinking reduced leaching, peroxide release and toxicity simultaneously [70]. Much of the toxicity reported at high substitution may therefore be an artefact of unbound catechol and residual coupling by-products rather than an intrinsic ceiling on substitution. Covalent immobilization of the catechol donor, selection of oxidation-resistant donors, and purification stringency should consequently be reported alongside substitution degree, which is rarely done and is a principal reason why cytotoxicity data for nominally similar conjugates are difficult to reconcile.

**Figure 4:**
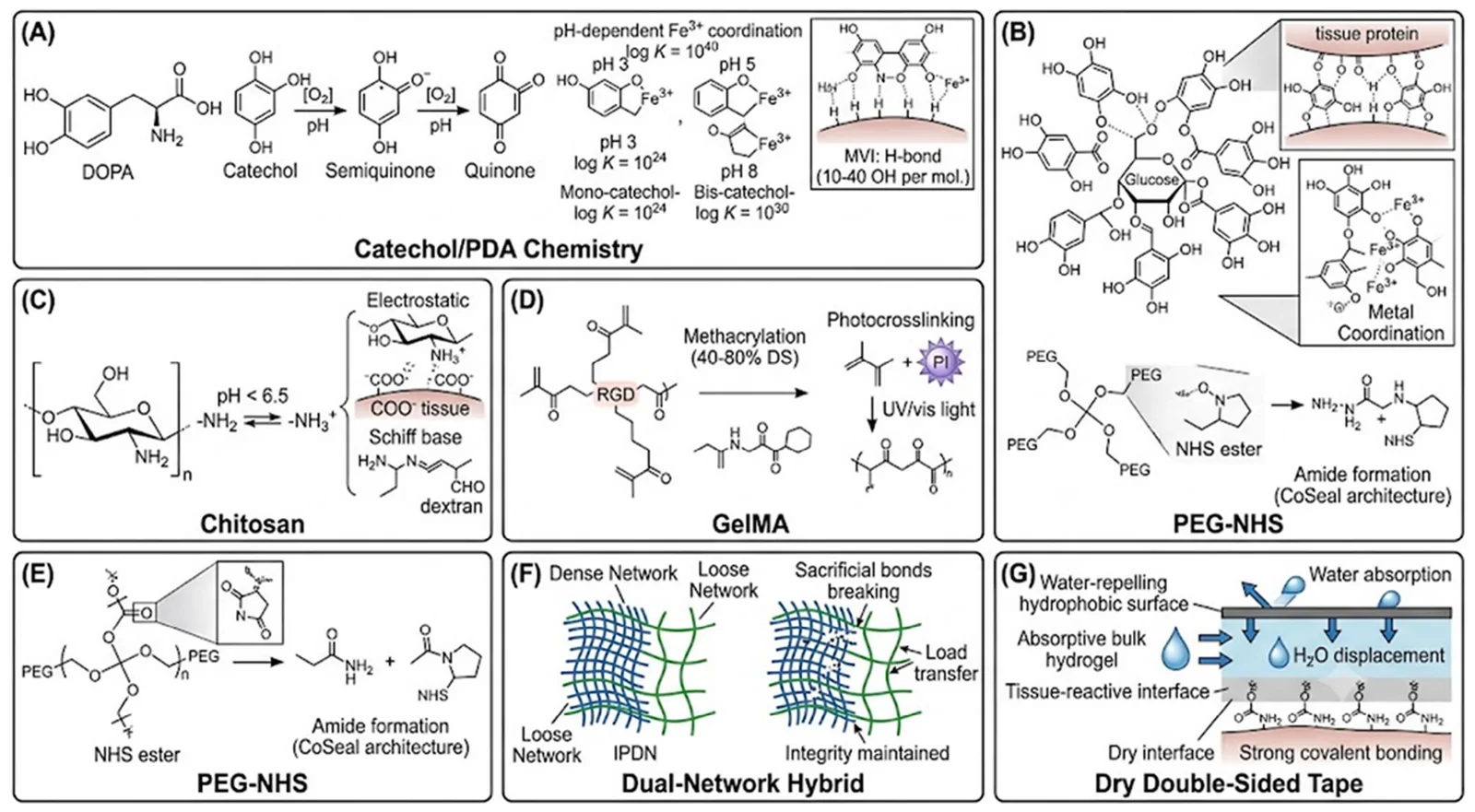
Representative material platforms and adhesion chemistries: (**A**) catechol/PDA—DOPA oxidation and pH-dependent Fe^3+^ coordination with tissue H-bonding; (**B**) TA—galloyl H-bonding and metal–phenolic coordination; (**C**) chitosan—electrostatic interaction and Schiff base formation below pH 6.5; (**D**) GelMA—methacrylated gelatinphotocrosslinked under UV/visible light; (**E**) PEG-NHS—amide bonding to tissue amines (CoSeal architecture); (**F**) dual-network hybrid—sacrificial bond rupture for energy dissipation and load transfer; and (**G**) dry double-sided tape—interfacial water displacement enabling rapid covalent bonding.

Oxidative polymerization of dopamine creates polydopamine coatings and nanoparticles with multivalent catechol presentation. PDA nanoparticle-reinforced hydrogels achieve enhanced adhesion through high catechol density, complemented by photothermal responsiveness for NIR-triggered antibacterial activity [71]. Han et al. developed mussel-inspired adhesive and tough hydrogels based on nanoclay-confined dopamine polymerization, achieving synergistic enhancement of adhesion through both catechol chemistry and nanocomposite reinforcement [72] (Figure 4A). The unresolved trade-off in this subcategory is reproducibility versus performance. The molecular structure of PDA is still contested—covalently linked dihydroxyindole and indoledione units versus non-covalent assemblies stabilized by hydrogen bonding and π-stacking [73]—and its formation is sensitive to oxygen availability, pH and ionic strength. In the absence of a defined structure, no critical quality attribute can be specified for batch release, which is a more immediate obstacle to translation than adhesion strength itself. The photothermal function is likewise more constrained than usually acknowledged, since NIR penetration of only a few millimetres restricts triggered antibacterial activity to superficial or endoscopically accessible sites, and the long-term fate of PDA nanoparticles retained at the implantation site remains poorly characterized.

TA’s abundance of phenolic hydroxyl groups (25 in the idealized decagalloyl glucose structure) creates multivalent adhesion and crosslinking sites far exceeding dopamine’s single catechol. TA-metal coordination (TA-Fe^3+^, TA-Cu^2+^) enables instant gelation with rapid strength development. TA–protein interactions (TA-gelatin, TA-collagen, TA-albumin) provide biocompatible adhesive networks. Shin et al. developed DNA/TA hybrid gels exhibiting biodegradability, extensibility, tissue adhesiveness, and hemostatic ability [37]. Fan et al. demonstrated TA-based multifunctional hydrogels with facile adjustable adhesion and cohesion through polyphenol supramolecular chemistry [39]. Bei et al. reviewed TA as a bioactive polyphenol for hydrogel engineering in wound healing; representative TA hydrogels combine adhesion with antibacterial (MIC of 25–30 μg/mL against *S. aureus* and *E. coli*), antioxidant (DPPH radical scavenging > 80%), anti-inflammatory, and hemostatic effects [41,74].

Two limitations offset this multivalency and are seldom stated explicitly. First, most TA-polymer networks are held together by hydrogen bonding and metal coordination rather than covalent bonds, so TA is extracted rapidly in aqueous media; the antibacterial and antioxidant activities cited above are consequently transient and are in part properties of the released TA rather than of the network, which confounds dose control and complicates the interpretation of MIC and DPPH values measured on bulk gels. Second, TA-Fe^3+^ coordination is itself pH-dependent, shifting between mono-, bis- and tris-complexes, so networks assembled under alkaline conditions partially disassemble in the acidic microenvironment of infected or inflamed tissue—the very setting in which these materials are proposed for use [75]. Commercial TA is moreover a heterogeneous mixture of galloyl glucose esters rather than a single compound, a plausible and under-reported contributor to the batch-to-batch variability discussed below.

However, catechol-based systems exhibit critical limitations that must be addressed for clinical translation. Catechol oxidation kinetics are highly sensitive to oxygen exposure, pH, and metal ion concentrations, creating batch-to-batch variability. Shelf-life stability is limited; catechol groups oxidize during storage, reducing adhesive capacity by 20–50% over 3–6 months even under a nitrogen atmosphere [72]. Fe^3+^ availability is limited under physiological conditions. High-affinity iron-binding proteins compete for it, with transferrin binding at a Kd near 10^−22^ M. In vivo adhesion may therefore fall below in vitro performance.

### 4.2. Chitosan-Based Systems

Chitosan, a deacetylated derivative of chitin (the second-most abundant polysaccharide in nature), exhibits inherent bioadhesive properties through electrostatic interactions with tissue proteins and mucopolysaccharides. The protonated amino groups on chitosan (pKa ~6.5) generate positive charges enabling mucoadhesion and electrostatic tissue interaction [76]. Two consequences of this mechanism are consistently underemphasized. First, electrostatic adhesion is short-range and is progressively screened at physiological ionic strength, so values obtained in water or dilute acidic buffer systematically overestimate performance in interstitial fluid or blood. Second, and more fundamentally, “chitosan” denotes a family of structurally distinct polymers rather than a single compound: molecular weight and degree of deacetylation (DD, typically 70–90% in commercial grades) jointly determine charge density, solubility, antimicrobial potency, degradation rate and immune response, yet much of the adhesive literature reports neither parameter, which makes the adhesion and antibacterial values collated below difficult to compare across studies [77,78].

Chitosan-alone hydrogels achieve modest adhesion (10–30 kPa), inadequate for demanding applications, but serve as excellent platforms for chemical modification and functional incorporation. Key chitosan derivative systems include:

Chitosan–catechol conjugates combine chitosan’s inherent adhesivity with dopamine or polyphenol functionality, achieving wet adhesion of 60–110 kPa, although this gain is inherited together with the catechol oxidation, shelf-life and quinone-toxicity liabilities detailed in Section 4.1. Carboxymethyl chitosan modifications increase water solubility at physiological pH while enabling stronger electrostatic interactions [79]. Oxidized polysaccharide crosslinkers, particularly oxidized dextran and oxidized hyaluronic acid, react with primary amines on chitosan to form Schiff base networks, enabling in situ gelation by simple mixing with rapid network formation (5–15 min) (Figure 4C). The convenience of this chemistry is inseparable from its principal weakness. Imine bonds remain in dynamic equilibrium with their amine and aldehyde precursors, and the equilibrium shifts towards hydrolysis under acidic conditions, so Schiff-base networks disintegrate in the acidic milieu of infected or ischaemic tissue while becoming mechanically weak at alkaline pH [80]. The crosslinker introduces a second, rarely discussed cost: periodate oxidation opens the sugar ring to generate the dialdehyde, which shortens the polysaccharide chains, abolishes the native bioactivity of hyaluronic acid, and leaves reactive aldehydes able to crosslink host proteins non-specifically. Residual periodate and unreacted aldehyde should therefore be quantified rather than assumed to be absent [80].

Quaternary ammonium chitosan (QAC) introduces permanent positive charges independent of pH, maintaining antimicrobial activity and tissue adhesion across the full physiological pH range. QAC-based adhesives achieve broad-spectrum antimicrobial efficacy (>90% killing against both Gram-positive and Gram-negative bacteria) through membrane disruption, without reliance on ion release or ROS generation [25]. Permanent cationic charge is not, however, a selective mechanism. The quaternary ammonium density and alkyl chain hydrophobicity that drive bacterial membrane disruption also govern haemolysis and mammalian cytotoxicity, so antimicrobial potency and biocompatibility scale together rather than independently, and the degree of substitution and alkyl chain length are as informative as the reported killing percentage [81]. Killing figures of this kind are moreover typically obtained against planktonic bacteria in low-ionic-strength broth over short contact times, whereas efficacy against established biofilms, and in the presence of serum proteins that neutralize cationic charge, is seldom reported.

Recent advances in chitosan adhesive systems include light-activated chitosan hydrogels achieving superior adhesion strength (31.4 ± 5.1 kPa) with broad-spectrum antibacterial efficacy (>98% killing against *E. coli* and *S. aureus*) via membrane disruption and ROS generation, with hemostasis achieved in 32 ± 3.6 s with 70% reduction in blood loss [82]. Researchers have developed immunomodulatory chitosan bioadhesive hydrogels for liver hemostasis and repair, demonstrating that chitosan may actively promote tissue regeneration beyond passive adhesion [83]. This interpretation warrants caution. Reported immune responses to chitosan are genuinely contradictory, encompassing both pro- and anti-inflammatory outcomes, and a substantial part of that divergence has been traced to endotoxin contamination rather than to chitosan itself: the polycationic backbone binds lipopolysaccharide avidly, commercial grades differ by orders of magnitude in endotoxin content, and dendritic cells respond to concentrations near 0.1 EU/mL—well below levels that most studies never measure [78]. Attributing a regenerative outcome to intrinsic immunomodulatory activity is therefore defensible only when endotoxin content has been quantified and reported, which is rarely the case.

Limitations of chitosan-based systems include pH-dependent solubility; chitosan dissolves only below pH 6, complicating physiological application, and potential immunogenicity of chitosan oligomers generated during degradation. Additionally, Schiff base crosslinking provides reversible bonds susceptible to hydrolysis, potentially reducing long-term adhesion durability. Glycol chitosan, a water-soluble chitosan derivative, addresses the solubility limitation yet maintains adhesive functionality [84]. These limitations are not independent, and the coupling between them defines the central design conflict of the platform. Raising DD improves solubility and increases cationic charge density, and hence adhesion and antimicrobial activity, but simultaneously suppresses lysozyme-mediated degradation, because lysozyme acts on the acetylated units, and increases the propensity for mitochondrial ROS generation and inflammasome activation [77,78]. Solubilising derivatisations such as carboxymethylation or glycol substitution resolve the pH constraint by consuming or shielding the very amino groups that mediate electrostatic adhesion, so the solubility gain is partly paid for in adhesive and antimicrobial performance—a trade-off that should be quantified rather than presented as a solution [81].

### 4.3. Gelatin/GelMA-Based Systems

Gelatin, derived from partial collagen hydrolysis, retains RGD cell-adhesion peptides and other bioactive sequences present in native ECM, providing inherent cell-instructive properties absent in synthetic polymers. Gelatin methacryloyl (GelMA), where methacrylate groups are covalently attached to gelatin lysine and tyrosine residues (degree of substitution typically 40–80%), enables photocrosslinking alongside the retention of bioactive functionality [85,86]. The stated substitution range should nonetheless be read with caution. Degree of functionalization is quantified by mutually inconsistent methods—free-amine assays such as TNBS or ninhydrin versus ^1^H NMR integration of the vinyl protons—which do not distinguish methacrylamide from methacrylate ester substitution and can differ substantially for the same material [87]. Combined with variation in gelatin source (porcine, bovine or fish skin), Bloom strength and molecular weight distribution, this means that formulations reported as nominally identical are frequently not comparable, and it is a principal reason why mechanical and adhesive values across the GelMA literature span such wide ranges [87,88].

GelMA adhesives achieve modest wet adhesion (20–40 kPa) through physical entanglement and topological interactions but benefit from excellent biocompatibility and natural ECM mimicry. This is a real but bounded advantage: at 20–40 kPa, GelMA alone is comparable to unmodified chitosan, and because adhesion arises predominantly from interdigitation rather than interfacial covalent bonding, it deteriorates sharply on wet, mucus-covered or actively bleeding surfaces where interpenetration is prevented—precisely the surfaces for which surgical adhesives are needed. The degree of methacrylation, GelMA concentration (5–20% *w*/*v*), and photoinitiator selection determine the resulting hydrogel’s mechanical properties, degradation rate, and cell response [7].

Photoinitiator selection in particular embodies an unresolved compromise. Irgacure 2959, still the most widely used initiator, absorbs poorly at wavelengths benign to cells and therefore requires UV exposure that is both mutagenic and strongly attenuated by tissue, whereas lithium phenyl-2,4,6-trimethylbenzoylphosphinate permits 405 nm visible-light curing at roughly one-tenth the gelation time with encapsulated-cell viability above 95% [89]. Neither system escapes oxygen inhibition of radical propagation, which is most severe precisely at the exposed adhesive-tissue boundary and leaves an under-cured interfacial layer; reporting bulk conversion or bulk modulus therefore says little about the strength of the interface that actually fails. GelMA functionalization with catechol groups (GelMA-DOPA) dramatically enhances adhesion to 70–100 kPa yet preserves cell-instructive properties. Montazerian et al. demonstrated stretchable and bioadhesive GelMA-based hydrogels enabled by in situ dopamine polymerization, achieving simultaneous mechanical robustness and strong tissue adhesion. Liang et al. developed paintable and rapidly bondable conductive GelMA-based hydrogels as therapeutic cardiac patches, achieving electrical conductivity matching cardiac tissue while maintaining adhesive integration [90] (Figure 4D). This gain is nonetheless obtained by importing catechol chemistry and, with it, the oxidative-stability and quinone-cytotoxicity constraints set out in Section 4.1; GelMA-DOPA is therefore better evaluated as a hybrid system inheriting two sets of limitations than as an incremental improvement on GelMA.

Recent work by Kim et al. developed photo-crosslinkable, injectable, and highly adhesive GelMA-glycol chitosan hydrogels specifically for cartilage repair [71,91,92], demonstrating the versatility of GelMA as a platform for tissue-specific adhesive optimization [84]. Photo-curable GelMA-chitosan bioadhesive hydrogels achieved rapid gelation (<30 s under visible light) with adhesion strengths suitable for intra-articular application. The suitability claim is, however, asserted rather than demonstrated. Cartilage presents a low-friction, proteoglycan-rich and comparatively amine-poor surface under cyclic compression and shear, so the informative endpoint is retention under physiological loading over weeks rather than the static lap-shear or pull-off strength these studies typically report.

GelMA photocrosslinking gives precise spatial control, so specific tissue regions can be sealed selectively under surgical visualisation. Crosslinking depth is nevertheless limited by light penetration. Recent enzymatic hydrolysis approaches remove thermal gelation while preserving photocrosslinkability, which supports high-concentration biofabrication with independently tunable stiffness [83]. Applications of GelMA-based adhesives emphasize cardiac, neural, cartilage, and vascular tissues where ECM mimicry and cell compatibility prove critical. Two further constraints temper the ECM-mimicry rationale that motivates these applications. Gelatin is an animal-derived, batch-variable raw material, imposing sourcing, viral-clearance and lot-qualification burdens that recombinant human gelatin resolves only at substantially higher cost. More fundamentally, the methacryloyl groups that enable crosslinking are installed on the lysine residues through which matrix metalloproteinases and cell-surface receptors engage the chain, so raising substitution simultaneously increases stiffness and adhesion while slowing enzymatic remodelling and reducing the density of accessible cell-binding motifs [88]. The bioactivity that distinguishes GelMA from synthetic adhesives is therefore consumed in proportion to the crosslinking that gives it mechanical function; this coupling, rather than light penetration depth, is the principal unresolved limitation of the platform.

### 4.4. PEG-Based Systems

PEG, synthesized through well-established pharmaceutical manufacturing processes, offers exceptional reliability, batch-to-batch consistency, and extensive regulatory precedent (FDA-approved for numerous parenteral and topical applications). PEG-based surgical sealants dominate the commercial adhesive market [3,93] (Figure 4B,E). It is worth stating explicitly that this commercial position reflects manufacturing reproducibility and an established regulatory pathway rather than adhesive performance: each product below is indicated as an adjunct to sutures or staples rather than as a replacement for them, and the efficacy figures reported for them describe sealing or leak prevention, not adhesion strength [16,67]:

Composed of 4-arm PEG–succinimidyl glutarate and 4-arm PEG–thiol, crosslinking with dilute HCl solution to rapidly form a covalently bonded hydrogel. CoSeal (Baxter International Inc.) achieves hemostatic efficacy of 86% in cardiovascular surgical applications, with adhesion to both tissue and synthetic graft materials. However, adhesion strength is modest (15–25 kPa), and swelling of 300–400% post-application limits use in confined spaces.

Composed of tetra-PEG-succinimidyl ester and trilysine amine, designed as an adjunct to sutured dural repair. DuraSeal (Medtronic) achieves watertight dural closure in 98.2% of cases but exhibits concerning post-application swelling of 50–400%, causing documented cases of spinal cord and nerve compression requiring revision surgery [25]. These are not isolated events: cord and cauda equina compression requiring reoperation has been reported across cervical, thoracic and lumbar applications, with expansion peaking three to fourteen days postoperatively and persisting for several weeks [94], and the manufacturer subsequently introduced a reduced-swelling variant intended specifically for spinal use. Researchers have developed low-swelling hydrogel alternatives for dural defect sealing, addressing this critical safety concern [54].

Swelling warrants more critical weight than it is normally given, because in PEG systems it is not a formulation defect that better processing could remove but a thermodynamic consequence of the design. Equilibrium swelling is set by the balance between the osmotic drive of a highly hydrophilic network and the elastic restoring force of its crosslinks, so the swelling ratio scales inversely with crosslink density—precisely the parameter that simultaneously governs gelation speed, cohesive strength and modulus. Raising crosslink density to suppress expansion therefore stiffens the sealant away from the compliance of the tissue to which it is bonded, whereas lowering it restores compliance at the cost of expansion. The 300–400% and 50–400% figures quoted above are best read as two positions on this single trade-off curve rather than as independent product characteristics.

Three consequences follow, none of which is captured by the endpoints against which these materials are routinely characterized. First, swelling degrades the adhesive interface itself: as the network imbibes water, it dilutes the areal density of polymer, and hence of interfacial bonds, at the contact plane, while the mismatch between a swelling adhesive and an essentially non-swelling tissue generates shear stress that promotes delamination. Because lap-shear and burst-pressure measurements are almost always performed immediately after curing, the 15–25 kPa reported for these systems describes the interface at its strongest and says little about its condition once equilibrium hydration is reached; adhesion measured before and after equilibrium swelling is rarely reported as a pair. Second, the quantity that matters in a confined anatomical space is not the free-swelling ratio of a specimen floating in buffer but the swelling pressure generated when expansion is mechanically constrained. Free swelling overstates the volume change that can actually occur in situ while understating the load transmitted to adjacent structures, and confined swelling pressure is essentially never reported in this literature. Third, the kinetics matter as much as the magnitude: expansion peaks days after implantation and persists for weeks, which places the failure mode outside both the intraoperative window in which sealing efficacy is judged and the 24–72 h window in which most preclinical adhesion retention is assessed.

Existing mitigation strategies confirm that this is a trade-off rather than a solved problem. Low-swelling formulations achieve their gains by changing the crosslinking chemistry outright rather than by optimizing an existing one—for example by replacing NHS-ester coupling with o-phthalaldehyde/amine condensation and introducing a protein co-network, which reduced the swelling ratio to roughly 33% against the 50–400% of commercial dural sealants while retaining adhesion of about 80 kPa [25]—or by restricting the applied volume to a thin layer, which shifts the burden onto surgeon technique and is poorly controlled in practice. A more immediately useful step for the field would be reporting convention: swelling ratio measured under confinement and at equilibrium, paired with adhesion strength measured at the same time point, would do more to make these systems comparable than further increments in peak adhesion.

PEG systems have a clear advantage in mechanical tunability. Molecular weight (3–40 kDa), arm number (2–8) and crosslink density together set the modulus, which ranges from soft (1–10 kPa) to stiff (>1000 kPa). PEG’s weak intrinsic adhesion (10–25 kPa) reflects its chemical inertness. Although this inertness is beneficial for biocompatibility, it necessitates enhancement through additional adhesion-promoting chemistry (catechol functionalization, adhesion peptides, NHS ester activation). Bu et al. developed Tetra-PEG-based hydrogel sealants achieving effective in vivo visceral hemostasis through optimized network topology providing both strong adhesion and mechanical compliance [93]. It should be noted that the modulus range quoted here and the swelling behaviour discussed above are controlled by the same variables, so the tunability presented as an advantage is bounded: a formulation cannot be independently optimized for compliance, cohesive strength and dimensional stability. Two residual liabilities deserve more attention than they currently receive. First, these networks are cleared by non-specific ester hydrolysis rather than by cell-mediated remodelling, so the degradation rate is fixed at synthesis and cannot adapt to the healing site; the inertness that confers biocompatibility is the same property that prevents the material from participating in repair.

Degradation and swelling are moreover coupled in the same direction: hydrolytic scission progressively lowers crosslink density, so a network that is dimensionally acceptable at implantation continues to expand as it degrades, and swelling is greatest in the period when cohesive strength is already falling. Second, anti-PEG IgG and IgM are detectable in a majority of the general population without prior exposure to PEGylated therapeutics [95]. Whether this pre-existing immunity affects locally implanted PEG hydrogels as it affects circulating PEGylated agents has not been systematically evaluated, and that absence of evidence is itself worth stating in a field that has treated PEG as immunologically inert. Taken together, the PEG platform illustrates the inverse of the catechol case: regulatory and manufacturing maturity are established, and the outstanding problems are material-intrinsic—weak interfacial bonding, swelling coupled to gelation kinetics and to degradation, and non-adaptive degradation—rather than translational.

### 4.5. Multi-Network and Hybrid Systems

Leading-edge bioadhesive development emphasizes multi-network and hybrid architectures combining complementary material properties. Dual-network hydrogels, typically comprising a stiff, highly crosslinked first network interpenetrated with a soft, sparsely crosslinked second network, achieve exceptional toughness through energy dissipation mechanisms; the stiff network sacrificially breaks under stress, with the soft network maintaining integrity and redistributing load [46,47].

Organic-inorganic hybrids add function through nanoparticles. Silica nanoparticles improve mechanical properties and bridge polymer chains to tissue surfaces. Hydroxyapatite nanoparticles add osteoconductivity for bone adhesion. Gold nanoparticles enable plasmonic photothermal effects. MXene (Ti_3_C_2_T_x_) nanosheets provide electrical conductivity together with antimicrobial activity [96,97]. Li et al. demonstrated that plant-inspired adhesive and tough hydrogels based on Ag-lignin nanoparticle-triggered dynamic redox catechol chemistry achieve synergistic enhancement of adhesion, toughness, and antibacterial activity [49].

GelMA-dopamine-chitosan triple networks represent the complexity frontier, incorporating ECM mimicry (GelMA), strong catechol adhesion (dopamine), and antimicrobial chitosan functionality. These systems exceed 80 kPa adhesion while also providing antibacterial and cell-instructive function. Manufacturing complexity, batch-to-batch variability and regulatory hurdles nevertheless limit their translation [47].

The increasing complexity of advanced systems must be weighed against manufacturing scalability, regulatory approval pathways (each additional component adds regulatory burden), and clinical cost-effectiveness. Materials combining no more than 2–3 components with well-established safety profiles represent the most realistic near-term clinical translation candidates.

One emerging class falls outside this comparison and warrants explicit mention, because it is advancing rapidly in the biological literature while remaining absent from clinical evaluation. Hydrogels built from genetically encoded proteins specify crosslink stoichiometry and biological function at the level of the gene rather than by post-synthetic modification. The SpyTag/SpyCatcher isopeptide pair assembles protein networks in physiological buffer without crosslinker, catalyst or photoinitiator, and allows cell-adhesive motifs, enzymes or growth-factor mimetics to be genetically fused into the network rather than entrapped within it [98,99]; crosslink architecture is itself a design variable, since varying SpyCatcher multiplicity per network node alters mesh topology and thereby the catalytic output of enzymes displayed in the gel [100]. Sequence-encoded dynamic crosslinks extend the same logic to self-healing, with histidine-rich coiled-coil motifs coordinated by Zn^2+^ setting stiffness and healing rate at constant polymer concentration [101]. In parallel, recombinant adhesive proteins pursue the biological adhesives of Section 2.4 directly: mussel foot protein Pvfp-5β [102], tissue-selective mussel-inspired underwater adhesive [103], body-temperature-activated protein adhesives carrying a decellularized matrix component [104], barnacle cement protein MrCP20 with intrinsic control of mineralization [105], elastin-like fusion designs addressing scalability [106], and elastin-like polypeptide–hyaluronic acid hybrids [107]. Reported wet adhesion, mostly 10–100 kPa, remains below that of the catechol hybrids, and no system in this class has entered clinical use; its distinguishing merit is exact molecular definition and genetically programmable bioactivity, and its bottleneck is recombinant yield, endotoxin control and cost of goods rather than adhesion strength. We therefore regard it as complementary to, rather than competing with, the chemically crosslinked platforms compared above, and as the most likely source of reproducible, precisely functionalized adhesives over the coming decade.

Our laboratory’s experience across several platforms points to one conclusion. Platform choice should be driven by the mechanical environment of the target tissue, not by adhesion strength alone. For static tissues (bone, dural), PEG-based systems with predictable gelation kinetics offer practical advantages despite modest adhesion. For dynamic tissues (cardiac, vocal fold, vascular), catechol-hybrid systems with self-healing capability are more appropriate despite greater synthetic complexity. This tissue-first design philosophy, rather than the material-first approach prevalent in the literature, may accelerate clinical translation by better matching material capabilities to clinical needs.

### 4.6. Critical Material Platform Comparison

Material platform selection depends critically on application requirements (Table 2). For wound healing and hemostasis, catechol/polyphenol systems (70–150 kPa) offer superior wet adhesion, natural antimicrobial properties, and self-healing capability, now supported by in vivo data showing 40–60% faster wound closure vs. fibrin sealants [40,43,97]. Cardiac and neural applications require electrical conductivity. Hybrid systems with MXenes or conductive polymers meet this need. Conductive GelMA-PEDOT derivatives reach 0.1–1 S/cm and combine adhesion with bioelectronic signal transmission [28,108,109,110]. For FDA-approved clinical translation, PEG-based systems (CoSeal, DuraSeal) provide regulatory precedent despite inferior self-healing (20–40% vs. 75–95% for catechol systems), explaining their continued dominance in dural sealing despite mechanical limitations.

The quantitative evidence favours dual-network and hybrid approaches over single-mechanism platforms. They reach 120–180 kPa adhesion with 75–95% self-healing and higher toughness. More than 50 preclinical in vivo studies across at least five tissue types now support this. However, these systems face reproducibility challenges during scale-up, longer gelation times (5–15 min vs. 30–90 s for PEG systems), and higher manufacturing costs—the primary barriers preventing broader clinical adoption. Future commercial development will likely follow two tracks. Commercial development will likely follow two tracks. The first is optimisation of hybrid systems for regulatory approval under rigorous GMP manufacturing. The second is continued niche use of PEG systems in existing clinical workflows, until superior next-generation alternatives are approved.

## 5. Functional Integration Beyond Simple Adhesion

The clinical value of bioadhesive hydrogels extends far beyond mechanical tissue bonding. Modern bioadhesive platforms integrate multiple therapeutic functions within a single material, creating comprehensive treatment systems that address the complex, multifaceted nature of tissue damage and repair.

### 5.1. Antibacterial Properties

Surgical site infections affect approximately 2–5% of surgical patients and 7–10% of traumatic wounds, with associated healthcare costs exceeding USD 3.3 billion annually in the United States alone [96]. Integration of antibacterial functionality into bioadhesive platforms addresses infection prevention directly at the wound site, avoiding systemic antibiotic administration and associated resistance concerns.

Silver nanoparticle (AgNP) incorporation remains the most extensively validated antimicrobial strategy. AgNPs generate antimicrobial effects through multiple mechanisms: silver ion release disrupting bacterial cell membranes, oxidative stress generation through catalytic ROS production, and direct nanomaterial–bacterial interaction [49]. Hydrogel-embedded AgNPs at concentrations of 0.1–2 μg/mL sustain antimicrobial activity over 2–4 weeks with minimal cytotoxicity to mammalian cells (>90% viability at antimicrobial concentrations). Qu et al. developed antibacterial adhesive injectable hydrogels with rapid self-healing, extensibility, and compressibility as wound dressings for joint skin wound healing [86].

Quaternary ammonium groups kill bacteria on contact by disrupting membranes, without releasing ions. This gives three advantages. Activity is sustained because the mechanism is not depletable. Efficacy is broad-spectrum across Gram-positive and Gram-negative bacteria. Cytotoxicity is lower than with heavy metals [97].

Some materials are inherently antibacterial and need no further modification. Chitosan disrupts bacterial membranes through electrostatic interactions with cell walls. TA acts through ROS generation and protein denaturation. PVA/ascorbic acid composites with TA synthesized by gamma irradiation demonstrated enhanced bioadhesive and antimicrobial properties with MIC values of 25 μg/mL and 30 μg/mL against *E. coli* and *S. aureus*, respectively [111].

**Figure 5:**
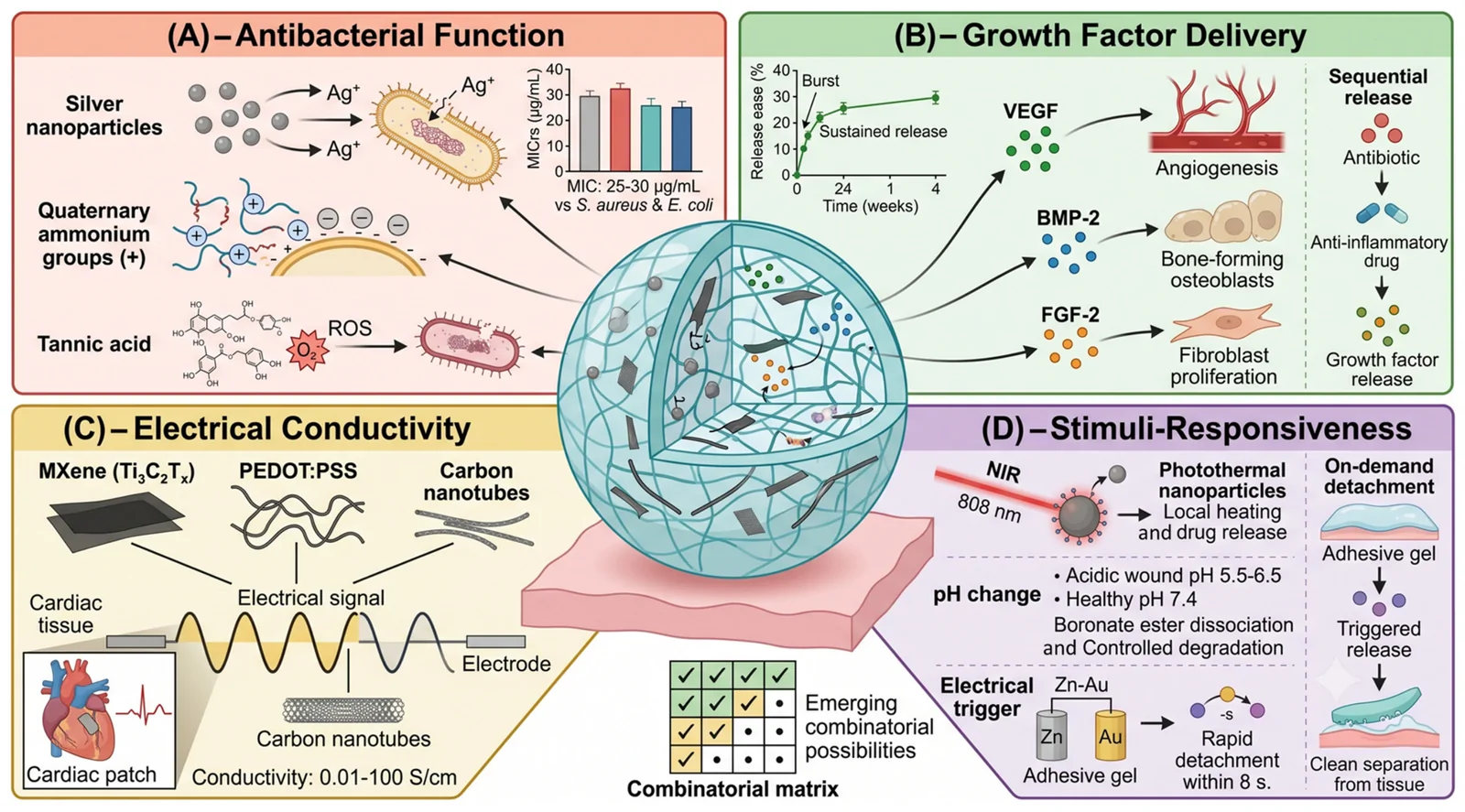
Multifunctional integration schematic: (**A**) antibacterial function (e.g., TA, peptide antibiotics, metal nanoparticles); (**B**) growth factor delivery (VEGF, bFGF, PDGF release kinetics); (**C**) electrical conductivity (conductive polymers, carbon nanotubes for cardiac/neural interfaces); and (**D**) stimuli-responsiveness (NIR/pH/glucose triggers for on-demand release or detachment).

Photothermal antibacterial strategies employ near-infrared-absorbing nanoparticles (gold nanorods, carbon nanotubes, MXene, polydopamine nanoparticles) generating localized heat (>50 °C) upon NIR illumination at 808 nm, rapidly killing bacteria while minimizing damage to surrounding tissue through careful power density control (0.5–2 W/cm^2^) [112]. Recent biomimetic 3D-printed adaptive hydrogel bioadhesives featuring combined chemical and photothermal antimicrobial mechanisms achieved superior infection resistance in contaminated wound models [31] (Figure 5A).

### 5.2. Drug and Growth Factor Delivery

Bioadhesive hydrogels function as ideal platforms for localized, sustained release of therapeutics including growth factors, antibiotics, anti-inflammatory agents, and analgesics. The hydrogel network can load drugs in four ways. Drugs may be physically encapsulated during gelation. They may form electrostatic complexes with charged polymer domains. They may be conjugated covalently to the backbone through cleavable linkers. They may also be carried in nanoparticles embedded in the matrix [108,113].

Four growth factors dominate these applications. Vascular endothelial growth factor (VEGF, effective at 0.1–10 ng/mL) promotes the angiogenesis needed for wound healing. Bone morphogenetic proteins, particularly BMP-2 at 0.1–1 μg/mL and BMP-7, enhance osteogenic differentiation and bone repair. Fibroblast growth factor (FGF-2, 1–100 ng/mL) promotes fibroblast proliferation and ECM deposition. Transforming growth factor-beta (TGF-β, 1–10 ng/mL) directs chondrogenic differentiation for cartilage repair [114].

Release kinetics from hydrogel systems are controlled through network crosslink density, hydrophobic domains sequestering hydrophobic drugs, degradation-mediated release, and affinity-based retention using heparin-binding domains or specific aptamers. Typical release profiles exhibit initial burst release (5–30% in the first 24 h) followed by sustained release over 1–4 weeks. Sequential release strategies employ multiple drugs with differing release kinetics: initial antibiotic burst preventing infection (day 0–3), followed by anti-inflammatory agent release reducing acute inflammation (day 1–7), followed by sustained growth factor release promoting tissue regeneration (week 1–4) [115].

Our laboratory developed growth factor-releasing bioadhesive scaffolds for vocal fold tissue engineering. They outperformed the adhesive alone and maintained growth factor bioactivity throughout the release period [14]. Four considerations are critical. Protein denaturation during hydrogel preparation must be avoided, which mild gelation conditions achieve. Bioactivity must be preserved during storage and release. Bioeffective concentrations must be reached at the target site. Off-target systemic exposure must be minimized (Figure 5B).

### 5.3. Electrical Conductivity

Cardiac and neural tissues are intrinsically electrically active, requiring bioelectronic interfaces that simultaneously provide mechanical adhesion and electrical conductivity for signal transmission across repair sites. Conductive adhesives incorporate conductive polymers (PEDOT:PSS, polyaniline, polypyrrole), carbon allotropes (graphene, carbon nanotubes, reduced graphene oxide), MXene nanosheets, or metal nanoparticles (gold, silver), achieving electrical conductivity of 0.01–100 S/cm depending on filler content and network structure [8,109].

Cardiac patch applications represent the most advanced conductive adhesive application. Lee et al. developed a conductive adhesive hydrogel cardiac patch combining MXene (Ti_3_C_2_T_x_) with gelatin and dextran aldehyde, achieving electrical conductivity of 18.3 mS/cm (similar to native cardiac tissue at ~10 mS/cm), elasticity of 30.4 kPa resembling cardiac tissue compliance, and strong tissue adhesion of 6.8 kPa [84]. A paintable and adhesive hydrogel cardiac patch with sustained ANGPTL4 release for infarcted heart repair demonstrated dual electrical and biochemical therapeutic functionality [116]. Zhao et al. (2025) comprehensively reviewed adhesive and conductive hydrogels for myocardial infarction treatment, establishing design criteria including matching native cardiac tissue conductivity, elasticity, and adhesion requirements [47].

Neural tissue applications include Deng et al.’s electrical bioadhesive interface achieving simultaneous mechanical adhesion and low-impedance electrical connection for long-term bioelectronic integration [8]. A highly stable, injectable, conductive hydrogel for chronic neuromodulation was recently demonstrated in Nature Communications, achieving mechanical and electrical stability for vagus nerve stimulation in a myocardial infarction therapy model [117]. Brain–computer interfaces represent an exciting future direction where adhesive coupling interfaces simultaneously provide mechanical integration, electrical signal transmission, and chemical biocompatibility, preventing glial scar formation (Figure 5C).

### 5.4. Self-Healing Mechanisms and Long-Term Durability

Beyond adhesion mechanisms discussed in Section 3.2, self-healing capabilities at the adhesive-tissue interface specifically extend functional longevity. Dynamic covalent bonds enable autonomous repair of interface damage through reversible breaking and reformation. Advanced designs employ dual-healing strategies: rapid dynamic healing (seconds to minutes via hydrogen bonds and metal coordination) for minor interfacial damage, and slower permanent crosslink formation (hours to days via covalent exchange) for sustained strength restoration [49,118].

Luo et al. developed a highly stretchable, real-time self-healable hydrogel adhesive matrix that recovered 95% of its strength within 2 min. Cooperative hydrogen bonding and metal coordination drove the repair. Multi-mechanism designs can therefore approach complete autonomous healing [109].

Standardized protocols are needed to quantify healing efficiency and long-term durability. These should include cyclic mechanical testing over thousands of physiologically relevant cycles. They should also include accelerated aging at elevated temperature and humidity. Finally, in vivo assessment should compare adhesion at implantation with adhesion weeks or months later. These standardization areas remain incomplete in the current literature.

### 5.5. Stimuli-Responsive and Smart Adhesion

Smart/stimuli-responsive adhesives represent a major frontier enabling dynamic adhesion control [119]. These materials modulate adhesion strength, drug release, degradation, or other properties in response to environmental triggers relevant to wound healing and disease [111].

pH-responsive systems exploit the acidic microenvironment of wounds (pH 5.5–6.5 in chronic wounds versus pH 7.4 in healthy tissue) and tumors (pH 6.5–7.0). Boronate ester crosslinks, stable at physiological pH but dissociating under acidic conditions, enable wound-environment-activated drug release and adhesion modulation [119].

Temperature-responsive adhesion enables reversible attachment/detachment through LCST transitions. A thermo-switchable underwater adhesive based on a Janus hydrogel design achieved over 1000-fold difference in adhesion strength between adhesive (37 °C) and non-adhesive (4 °C) states, enabling strong adhesion during application followed by gentle removal through cooling [59]. Enzyme-responsive adhesives activated by wound-associated enzymes (MMPs, hyaluronidase, elastase) provide disease-specific responsive behavior. MMP-cleavable crosslinks enable accelerated degradation in inflamed tissue environments, releasing encapsulated therapeutics precisely where needed [58]. On-demand detachment represents an underexplored but clinically significant frontier. Adhesives that debond upon specific triggers, reductant-mediated disulfide cleavage, light-induced bond scission, and electrical field-triggered release (demonstrated within 8 s using Zn-Au electric fields), enable controlled removal when adhesion is no longer needed, preventing secondary tissue damage during dressing changes [38,42].

Our group’s work on vocal fold bioadhesives has revealed that soft tissue sealing applications present fundamentally different challenges from wound closure: the adhesive must maintain integrity under continuous dynamic loading without compromising the delicate mucosal wave propagation essential for phonation. This requires not only adequate adhesion strength but also precise viscoelastic matching, a design parameter rarely addressed in the broader bioadhesive literature but critical for functional tissue restoration [14] (Figure 5D).

## 6. Applications by Tissue Type

### 6.1. Wound Healing and Skin Regeneration

Acute and chronic skin wounds affect hundreds of millions of patients annually, with chronic wounds alone costing the global healthcare system over USD 28 billion per year. Current dressing standards (gauze, hydrocolloids, foam dressings) passively manage wound environments without actively promoting healing [120].

Hemostatic wound dressings incorporating coagulation-promoting factors (thrombin, calcium chloride) or topological adhesion features achieve rapid bleeding cessation critical for trauma and surgical applications. Shin et al. demonstrated complete prevention of blood loss with self-sealing hemostatic needles incorporating bioadhesive coatings [115]. Injectable self-expanding/self-propelling hydrogel adhesives with procoagulant activity and rapid gelation were developed for lethal massive hemorrhage management, achieving hemostasis within 30 s in liver and femoral artery injury models [121]. A recently developed rapidly photocurable and strongly adhesive hydrogel-based sealant demonstrated excellent procoagulant activity for lethal hemorrhage control with gelation under 10 s [122].

Multifunctional wound dressings simultaneously address multiple wound healing barriers. Ma et al. developed liquid bandage systems harvesting robust adhesive, hemostatic, and antibacterial performances as first-aid tissue adhesives [113]. Guo et al. demonstrated snake extract-laden hemostatic bioadhesive gels crosslinked by visible light, achieving enhanced hemostasis through biological coagulation activation [108]. Zhao et al. developed physical double-network hydrogel adhesives with rapid shape adaptability, fast self-healing, antioxidant and NIR/pH stimulus-responsiveness for multidrug-resistant bacterial infection and removable wound dressing [83].

Chronic wound applications particularly benefit from bioadhesive integration. Diabetic ulcers exhibit impaired healing due to reduced angiogenesis, altered growth factor signaling, and chronic infection. Multi-component hydrogels integrating anti-infective glycyrrhizic acid with self-healing and adhesive properties demonstrated accelerated acute wound healing and tissue regeneration. Poly(lipoic acid-co-sodium lipoate)-phytic acid hydrogels with disulfide and hydrogen bond crosslinking achieved 95% antibacterial efficacy in infected wound models while promoting epithelialization and collagen deposition [74].

Wearable wound monitoring integration represents an emerging direction. Strain-sensitive adhesive hydrogels enable real-time wound deformation monitoring, complemented by embedded biosensors that detect pH changes, temperature fluctuations, and biomarkers indicative of infection or healing progression [123] (Figure 1d).

### 6.2. Bone and Cartilage Repair

Bone fractures and cartilage injuries represent major orthopedic challenges, with adhesive approaches offering unique advantages over traditional fixation methods [124].

Bone adhesive applications: Injectable bioadhesive bone fillers conform to complex defect geometries, solidify in situ to form load-bearing constructs, and incorporate osteogenic factors and mineral phases. Catechol-functionalized adhesive scaffolds provide strong mineral integration through divalent cation (Ca^2+^, Mg^2+^) coordination, mimicking natural biomineralization. Li et al. developed ROS-responsive hydrogel coatings modified with titanium that promote vascularization and osteointegration of bone defects by orchestrating immunomodulation. Dual-network architectures or mineral-reinforced composites achieve compressive moduli of 100–1000 kPa approaching cancellous bone, though tensile strength matching cortical bone remains challenging.

Cartilage repair presents unique challenges due to the avascular nature, limited intrinsic healing capacity, and low-adhesion surface of articular cartilage. Recent work reported an immune-modulated adhesive hydrogel (PHE-Gel) for osteochondral graft fixation and cartilage repair. It improved the outcome of osteochondral autograft transfer by forming a stable adhesive interface between graft and host tissue [125]. A dual adhesive approach addressed the two different requirements of the osteochondral interface in a preclinical goat model. A phosphoserine-modified calcium phosphate cement bonded bone, and a methacrylated phosphoserine-containing gelatin hydrogel bonded cartilage [126].

Osteochondral interface repair requires gradient materials transitioning from hard mineral phase (bone) to soft hydrogel (cartilage). Triphasic hydrogel scaffolds incorporating an intermediate mineralized layer simulating the calcified cartilage zone facilitate mechanical integration and prevent cartilage ossification. Double-network bilayer hydrogels loaded with bioactive molecules (puerarin, curcumin) for osteochondral repair demonstrated zone-specific therapeutic delivery through stratified scaffold architecture [127] (Figure 1d).

### 6.3. Soft Tissue Sealing (Vocal Fold, Dural, Oral)

Soft tissue sealing applications require adhesives combining compliance, biocompatibility, and robust wet adhesion on delicate tissue surfaces.

Vocal fold repair: The larynx and vocal folds represent a critical application domain where bioadhesive hydrogels uniquely address unmet clinical needs. Vocal fold injuries result in voice loss and aspiration risk, affecting quality of life. Bioadhesive hydrogels offer injectable formulations conforming to vocal fold contours, elasticity supporting phonation (elastic modulus 1–10 kPa), promotion of tissue healing through growth factor delivery, and minimal invasiveness. Our laboratory developed self-healing bioadhesive hydrogels for vocal fold tissue implants, demonstrating mucosal integration, maintained elasticity, and functional voice recovery in animal models [14]. Four design parameters are critical: elasticity that permits natural vibration; adhesion of 20–50 kPa to bear load during phonation; self-healing that tolerates repeated stress at 100–300 Hz; and low immunogenicity to avoid granulation tissue.

Dural repair: A tough bioadhesive hydrogel for sutureless sealing of the dural membrane was demonstrated in porcine and ex vivo human tissu. This dural tough adhesive (DTA) exhibited greater toughness and higher maximum stress compared to commercial sealants in aqueous environments, with biocompatibility confirmed over 4 weeks in a rat craniotomy model [128]. This addresses the clinical limitation of DuraSeal’s excessive swelling by employing a tough hydrogel design that maintains volume stability post-application.

Oral wound closure: Hydrogels as bioadhesive materials for sutureless oral wound closure face the extreme challenge of adhesion in the persistently wet, enzyme-rich, mechanically dynamic oral environment. Recent evaluation established that effective oral bioadhesives require resistance to salivary enzymes, tolerance of pH fluctuations (5.5–7.5), and maintenance of adhesion during mastication forces (Figure 1d).

### 6.4. Vascular Repair and Hemostasis

Hemostatic tissue sealants preventing bleeding from surgical sites and traumatic wounds are critical for survival in hemorrhagic shock. Bioadhesive hydrogels incorporating hemostatic factors (thrombin, calcium chloride) and structural components provide multifunctional hemostasis [32].

Hong et al. developed a strongly adhesive hemostatic hydrogel for arterial and cardiac bleeding. It resisted burst pressures above 300 mmHg, which is sufficient for arterial pressure. Catechol-mediated tissue adhesion was combined with a physically tough hydrogel network. This represented a significant advance over fibrin glue (10–20 mmHg burst pressure) and commercial sealants (typically 40–100 mmHg). The development of adhesive hydrogels specifically engineered for hemostasis and vascular repair has been comprehensively reviewed [121], highlighting strategies including injectable hemostatic hydrogels for incompressible bleeding from deep internal injuries.

Vascular graft integration represents a distinct challenge where bioadhesive coatings on synthetic grafts reduce thrombogenicity, promote endothelialization, and enhance graft-to-vessel adhesion. Catechol-based coatings demonstrate reduced platelet adhesion, coupled with the promotion of endothelial cell attachment. Anti-thrombogenic adhesive coatings achieved through heparin incorporation maintain hemostatic function yet effectively prevent pathological thrombosis. Zhu et al. developed a novel DOPA-albumin-based tissue adhesive for internal medical applications, achieving strong adhesion to vascular tissue and exceptional blood compatibility [129] (Figure 1d).

### 6.5. Neural Tissue Repair and Bioelectronic Interfaces

Peripheral nerve injuries affecting millions annually result in permanent disability without effective regeneration strategies. Adhesive nerve conduits bonded to nerve stumps create guidance channels promoting axonal regrowth with advantages of conformal fit and reduced tissue trauma compared to microsuturing.

Conductive adhesive hydrogels for neural tissue represent an emerging frontier [63]. Electrical stimulation (10–100 mV/mm) activates voltage-gated ion channels, increases intracellular calcium, and enhances neurotrophic factor expression, all promoting neural regeneration. Adhesive interfaces maintaining electrical conductivity across tissue repair sites enable external stimulation without direct electrode implantation [8]. Highly stable, injectable, conductive hydrogels were recently demonstrated for chronic neuromodulation, achieving minimal tissue damage with low and stable impedance for vagus nerve stimulation [117].

Brain–computer interfaces are a promising future direction. An adhesive coupling interface must do three things at once. It must integrate mechanically with neural tissue, matching its elastic modulus of about 1 kPa. It must transmit electrical signals for high-fidelity recording and stimulation. It must also remain chemically biocompatible, so that glial scarring is prevented [130]. Bioadhesive hydrogel-coupled and miniaturized ultrasound transducer systems for long-term wearable neuromodulation recent studies demonstrate, highlighting the convergence of adhesive materials with bioelectronic devices.

Spinal cord injury offers limited natural recovery and remains devastating. Adhesive scaffolds address it by combining mechanical support, electrical stimulation, growth factor delivery and neural cell transplantation in a single platform (Table 3) (Figure 1d).

### 6.6. Comparative Clinical Readiness Across Tissue Applications

Section 6.1, Section 6.2, Section 6.3, Section 6.4 and Section 6.5 show that bioadhesive hydrogels have been demonstrated in essentially every tissue repair setting, but not that those settings are equally close to clinical use. Table 4 compares them on four common axes: current clinical readiness, best-matched platform, principal remaining challenge, and the most immediate opportunity.

Two patterns emerge. First, readiness tracks the mechanical simplicity of the target site rather than the sophistication of the material: the approved indications are those in which the adhesive seals a comparatively static surface, whereas every indication requiring sustained function under cyclic deformation—cardiac, vocal fold, vascular anastomosis—remains preclinical despite strong reported adhesion. Second, the remaining obstacles are application-specific and largely orthogonal to adhesion strength: swelling in dural sealing, mineral-interface integrity in bone, cyclic-strain durability in cardiac repair, and long-term impedance stability at neural interfaces. Adhesion strength is therefore rarely the limiting parameter, which motivates the translational analysis of Section 7.

## 7. Conclusions

Bioadhesive hydrogels have progressed from passive mechanical fixation toward multifunctional platforms engineered against tissue-specific requirements, combining physical, chemical, topological, and bioinspired mechanisms within single materials. This progress is, however, unevenly distributed with respect to clinical use. Only a limited set of chemistries is approved and in routine practice—PEG-NHS sealants and fibrin in vascular sealing and dural repair, and fibrin and cyanoacrylate benchmarks in skin closure—whereas gastrointestinal, bone and cartilage, cardiac, vocal fold and neural applications remain preclinical, with vocal fold and neural interfaces at an early preclinical stage (Table 4). Reported wet adhesion falls largely between 40 and 200 kPa across the platforms compared here, with individual systems reaching approximately 1800 kPa tensile strength on wet porcine skin [39] (Table 2). These figures are best read as indicative rather than directly comparable, since differences in tissue source, surface preparation and test geometry generate 5–10-fold variation in values reported for the same adhesive, and no standardized wet-adhesion protocol is yet in general use (Section 3.3). Multifunctional capabilities such as antibacterial activity, growth factor delivery, electrical conductivity, and stimuli-responsive behaviour have likewise been demonstrated individually and in combination, though predominantly in acute animal models and over observation periods shorter than the intended in situ lifetime of the material. Consistent with the platform comparison of Section 4.6, no single adhesion strategy emerges as optimal across applications: the appropriate choice is set by the mechanical and biological demands of the target tissue and, for near-term translation, by manufacturing and regulatory considerations at least as much as by adhesion strength.

Despite this scientific progress, the field has remained “on the verge of clinical translation” for over a decade, in large part because of systemic rather than purely scientific obstacles. One recurring hurdle is the absence of standardized adhesion testing protocols, which currently yields 5-to-10-fold variation for identical materials and limits cross-study comparability. Clinical translation is further constrained by ambiguous regulatory classification, manufacturing scalability limits, and the difficulty of maintaining shelf-life stability for highly reactive chemical components. Academic incentives appear to have favoured novel material design over rigorous preclinical validation, which offers a plausible explanation for the large number of published formulations relative to the number entering clinical evaluation as tissue adhesives, even though more than 400 clinical trials involving hydrogel materials are ongoing across all indications and 28 injectable hydrogel products had received FDA or EMA approval as of 2024 [11]. Narrowing this gap will likely require sustained investment in the less visible work of manufacturing optimization, long-term stability testing, and adequately powered large-animal preclinical studies.

Several developments may shape the next phase of the field, although their eventual impact remains to be established. The convergence of advanced chemistry, bioelectronics, and 3D bioprinting broadens the accessible design space, and data-driven design has recently been demonstrated in this context: Liao et al. reported de novo discovery of strongly adhesive hydrogels by machine learning over a defined compositional space [13]. Whether such approaches generalize beyond the searched space, and whether the resulting compositions also satisfy biocompatibility, degradation and manufacturability constraints, has yet to be shown. On-demand detachable adhesives, which respond to chemical, physical, or enzymatic triggers, and personalized, patient-specific 3D-printed bioadhesives address real surgical needs but remain at an early stage of validation. What can be stated with greater confidence is what translation would require: adhesion testing protocols that are standardized and fully reported; large-animal studies powered for retention and mode-of-failure endpoints rather than acute strength alone; and processes compatible with GMP manufacture and realistic shelf life. Progress on these fronts, rather than further increases in reported adhesion strength, will likely determine how many of the systems reviewed here reach clinical use.

## Figures and Tables

**Table 1:** Adhesion strength requirements and commercial benchmarks by tissue type.

Application	Required Shear Strength (kPa)	Burst Pressure (mmHg)	Key Mechanical Demands	Commercial Benchmark	Benchmark Performance
Skin wound closure	10–30	N/A	Flexibility, conformability	Fibrin glue (Tisseel)	~10 kPa wet
Bone/cartilage repair	50–200	N/A	Compressive strength, osteointegration	Cyanoacrylate	~100 kPa (brittle)
Vascular sealing	20–80	100–300	Elasticity, hemostasis	CoSeal (PEG-NHS)	15–25 kPa
Dural sealing	15–50	40–80	Low swelling, neural compatibility	DuraSeal	98.2% efficacy, but 50–400% swelling
Cardiac patch	30–100	N/A	Cyclic strain tolerance (10–20%, 1–2 Hz)	None approved	Experimental: 50–120 kPa
Vocal fold repair	5–30	N/A	Ultra-soft modulus (0.1–1 kPa), vibration resistance	None approved	Experimental only
Neural tissue	1–10	N/A	Ultra-soft modulus matching brain (~0.5 kPa)	None approved	Experimental only
Hemostatic emergency	10–50	>120	Rapid gelation (<30 s), blood compatibility	QuikClot (kaolin)	Not hydrogel-based

**Table 2:** Comparative summary of major bioadhesive hydrogel platforms.

Material Platform	Adhesion Mechanism	Wet Adhesion Strength (kPa)	Self-Healing (% Recovery)	Key Advantages	Key Limitations	References
Catechol/PDA	Catechol–metal coordination + hydrogen bonding	80–150	60–90	High wet strength, tunable chemistry, versatile modifications	pH-dependent, potential dopamine toxicity	[17,39,42]
TA	Polyphenol interactions + hydrogen bonding	0–120	70–85	Natural source, antimicrobial, biocompatible, cost-effective	Limited mechanical properties, slower gelation	[44,45,47,48]
Chitosan	Electrostatic + hydrogen bonding	40–80	50–70	Biocompatible, biodegradable, hemostatic, natural	Moderate adhesion strength, pH-sensitive	[75,83,84,85,86]
Chitosan-catechol	Catechol–metal + electrostatic interactions	90–140	75–88	Enhanced adhesion, improved mechanics, multifunctional	Synthesis complexity, higher cost	[75,76,83]
GelMA	Methacrylate crosslinking + cell interaction	50–100	40–65	Cell-compatible, tunable properties, photo-crosslinkable	Lower wet adhesion, photoinitiator toxicity	[85,87,89,94]
GelMA-DOPA	GelMA + dopamine integration	110–160	75–90	Combined mechanics + adhesion, excellent biocompatibility	Complex synthesis, storage stability	[85]
PEG-NHS (CoSeal)	Nucleophilic addition + covalent crosslinking	60–100	20–40	FDA-approved, clinical use, rapid gelation	Brittle, limited self-healing, permanent	[27,60,95]
Dual-network hybrid	Multiple complementary mechanisms	120–180	75–95	Superior toughness, excellent self-healing, optimal mechanics	Complex synthesis, reproducibility, longer gelation	[13,33,51,96]
Dry double-sided tape	Water-repelling surface + absorptive bulk	100–200	Variable	Highest wet adhesion, reversible, minimal damage	Limited tissue integration, non-biodegradable	[8,31]

Note: Adhesion strengths and self-healing percentages represent typical experimental ranges from peer-reviewed literature (2017–2025). Wet adhesion values from lap-shear and tensile tests on wet tissue substrates. Self-healing percentages indicate mechanical property recovery after damage. GelMA = gelatin methacryloyl; DOPA = dihydroxyphenylalanine; PEG = polyethylene glycol; NHS = N-hydroxysuccinimide.

**Table 3:** Tissue-specific applications: representative systems and key in vivo outcomes.

Application	Representative System	Animal Model	Key Outcome	Healing Time	Ref.
Diabetic wound healing	Catechol–chitosan/TA-Fe^3+^	Rat (STZ diabetic)	95% wound closure, M2 polarization	14 days	[84]
Bone defect repair	GelMA-nHA composite	Rabbit cranial defect	85% bone filling (micro-CT)	12 weeks	[92]
Cartilage repair	HA-Dopa/PEG hydrogel	Rabbit osteochondral	Neo-cartilage formation, type II collagen+	8 weeks	[101]
Dural sealing	Low-swelling PEG hydrogel	Porcine dural defect	Zero CSF leakage, <5% swelling	4 weeks	[108]
Vascular hemostasis	QAC-catechol hydrogel	Rat liver/cardiac puncture	Hemostasis in <30 s, 70% blood loss reduction	Acute	[85]
Cardiac patch	MXene-conductive adhesive	Rat MI model	Improved EF by 15%, electrical coupling	4 weeks	[86]
Vocal fold repair	Self-healing bioadhesive	Preclinical	Maintained adhesion under cyclic vibration	Ongoing	[15]
Neural interface	Conducting polymer adhesive	Rat sciatic nerve	Nerve conduction recovery, bioelectronic signal	8 weeks	[9]
Gastric ulcer	pH-independent fast-gel	Porcine gastric	Complete ulcer healing in acidic environment	2 weeks	[62]

**Table 4:** Comparative clinical readiness, remaining challenges and near-term opportunities by tissue application.

Application (Section)	Clinical Readiness	Best-Matched Platform	Principal Remaining Challenge	Most Immediate Opportunity
Skin wound closure and dressing (Section 6.1)	Approved and in routine use (fibrin, cyanoacrylate benchmarks)	Catechol/TA and chitosan; physical double networks	Durability over joint movement; chronic-wound infection; atraumatic removal	Multifunctional dressings with on-demand detachment
Vascular sealing and hemostasis (Section 6.4)	Approved and in routine use (PEG-NHS sealants, fibrin)	Tetra-PEG sealants; rapid-gelling catechol systems	Burst-pressure retention under pulsatile pressure; swelling in confined spaces	Non-compressible and pre-hospital hemorrhage control
Dural sealing (Section 6.3)	Approved product available; validated in porcine and ex vivo human tissue	Low-swelling PEG; tough double-network adhesives	Swelling-induced neural compression; postoperative adhesion	Suture-free closure competing on swelling, not adhesion
Gastrointestinal and mucosal repair (Section 3.3 and Section 6.3)	Large-animal (porcine) preclinical	pH-independent ultrafast-gelling systems	Adhesion in gastric acid under peristalsis; endoscopic delivery	Endoscopic ulcer and perforation sealing
Bone and cartilage repair (Section 6.2)	Large-animal preclinical	Catechol–mineral composites; GelMA–nHA; osteochondral double networks	Load-bearing strength; wet mineralized interface; osteointegration timescale	Osteochondral fragment fixation as a low-load first indication
Cardiac patches (Section 5.3 and Section 6.4)	Small- to large-animal preclinical	Conductive MXene- or PEDOT-based adhesive hydrogels	Retention under 10–20% cyclic strain at 1–2 Hz; epicardial delivery; arrhythmogenic risk	Combined electrical and biochemical post-infarction patches
Vocal fold and neural interfaces (Section 6.3 and Section 6.5)	Early preclinical	Ultrasoft viscoelastically matched gels; injectable conductive hydrogels	Viscoelastic matching at ~0.5–1 kPa; long-term impedance stability; glial scarring	Phonation-preserving sealing; adhesive bioelectronic coupling

## Data Availability

No new data were created or analyzed in this study. Data sharing is not applicable to this article.
